# Tailored Stitching and Vertical Stacking for High‐Voltage Multifunctional Structural Batteries with Enhanced Electrochemical–Mechanical Coupling

**DOI:** 10.1002/advs.202514967

**Published:** 2025-10-21

**Authors:** Gilsu Park, Chun‐Gon Kim, Heechul Kwon, Jayden Dongwoo Lee, Jinwoo Park, Yoonkook Son, Jong Guk Kim, Ji‐Hun Cha

**Affiliations:** ^1^ Department of Aerospace Engineering Chosun University 309, Pilmun‐daero, Dong‐gu Gwangju 61452 Republic of Korea; ^2^ Department of Aerospace Engineering KAIST 291, Daehak‐ro, Yuseong‐gu Daejeon 34141 Republic of Korea; ^3^ School of Space Engineering Sciences Kyungpook National University 80, Daehak‐ro, Buk‐gu Daegu 41566 Republic of Korea; ^4^ Department of Electrical Engineering Chosun University 309, Pilmun‐daero, Dong‐gu Gwangju 61452 Republic of Korea; ^5^ Department of Advanced Energy Engineering Chosun University 309, Pilmun‐daero, Dong‐gu Gwangju 61452 Republic of Korea

**Keywords:** composites, interlaminar reinforcement, multilayer energy storage, stitch densities, thermoplastic matrix

## Abstract

Multifunctional structural batteries combining mechanical load‐bearing and energy storage offer strong potential for lightweight systems. However, conventional carbon‐fiber‐reinforced polymer (CFRP)‐based designs face persistent challenges including electrode delamination, inter‐cell dead space, poor electrolyte stability, and insufficient interfacial bonding. This study introduces a CFRP‐based, vertically stacked high‐voltage structural battery that integrates through‐thickness aramid fiber stitching with selectively structured thermoplastic interfaces. The system employs Elium resin and polypropylene barriers to enhance moisture and oxygen shielding while preserving mechanical integrity. Tailored stitching architectures are systematically investigated over a range of stitch densities, revealing their influence on both electrochemical impedance and interlaminar shear strength. The resulting structure exhibits an energy density of 42.2 Wh kg^−1^ based on total structural mass, representing a 14% improvement over the unstitched configuration. Notably, the stitched architecture yields a flexural strength of 215.6 MPa and a modulus of 14.7 GPa, representing a 40% enhancement in mechanical properties over unstitched counterparts. This level of performance places the system among the most competitive structural battery designs reported to date in terms of multifunctional integration. Energy performance remains stable under mechanical deformation, significantly outperforming unstitched configurations in both electrochemical and structural resilience.

## Graphical abstract

A stitched, vertically stacked CFRP structural battery uses Kevlar interlaminar threads and selective PP/Elium® thermoplastics to maintain tight electrode spacing and robust electrolyte wetting. The architecture sustains electrochemical performance under bending and delivers higher pack voltage via through‐thickness series connections without sacrificing laminate stiffness.

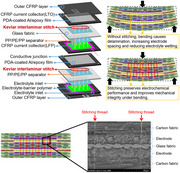

## 1 Introduction

Over the past several decades, the performance of electric vehicles (EVs), urban air mobility (UAM), unmanned aerial vehicles (UAVs), and satellites has significantly advanced. However, the development of lightweight batteries has not progressed at a comparable rate. As power demands for future mobility systems become more complex and high‐power electronic components continue to increase in number, the need for high‐capacity and lightweight energy storage solutions is expected to grow continuously.

Considering the current stagnation in battery development, the evolution of batteries into integrated structural components with lightweight and multifunctional capabilities appears to be a highly promising approach. Structural battery (SB) composites integrate energy storage functions directly into load‐bearing structures, thereby improving system‐level efficiency and reducing unnecessary mass. The concept of energy‐storing structural materials, in which batteries serve both structural and electrochemical functions, represents a transformative innovation for power management in EV, UAM, UAV, and satellites.

Carbon fiber‐reinforced polymer (CFRP) composites are widely explored in multifunctional energy storage systems because of their unique combination of load‐bearing capability and intrinsic electron transport characteristics. CFRP‐based structural batteries exhibit high mechanical strength, lightweight characteristics, and electrochemical stability, making them a leading candidate for practical multifunctional energy storage applications.

While various studies^[^ ^1^, ^2^ ^]^ have explored different structural battery configurations, this research focuses on laminated structural battery composites. Laminated structural batteries are generally produced as composite laminates through techniques including vacuum infusion processes or manual layering of pre‐impregnated fiber sheets. One of the critical benefits of laminated structural batteries with CFRP current collectors is the ability to tailor the mechanical performance while simultaneously enabling electrochemical functionality. The ability to control the thickness and stacking sequence of carbon fiber layers allows for optimization of structural integrity, leading to enhanced mechanical performance while ensuring the efficient integration of electrochemical components.^[^ ^1^ ^]^ Additionally, laminated composites allow for selective material placement, enabling complex structural designs that enhance multifunctionality and potential industrial scalability.

Several studies have demonstrated the feasibility of laminated structural battery composites, as summarized in Table S1 (Supporting Information).^[^ ^3^, ^4^, ^5^, ^6^, ^7^, ^8^, ^9^, ^10^ ^]^ While proof‐of‐concept designs have validated their potential, some key challenges remain. One of the major limitations is the oxygen and moisture permeability of structural polymer matrices. Most commonly used structural resins, such as epoxy, exhibit oxygen and water vapor permeability levels that are insufficient to protect lithium‐ion electrolytes, except for specialized polymers like polypropylene (PP).^[^ ^11^ ^]^ Consequently, some studies have proposed alternatives such as ionic liquid electrolytes,^[^ ^4^ ^]^ gel electrolytes,^[^ ^5^ ^]^ or ambient‐stable liquid electrolytes^[^ ^6^ ^]^ when integrating fiber‐reinforced composites with laminated battery structures. However, the long‐term environmental stability of the specialized electrolyte mentioned in the epoxy matrix barrier has not been comprehensively evaluated, leaving uncertainties regarding the retention of electrolyte performance under prolonged exposure to ambient conditions. Another issue with ionic or gel electrolytes is their lower ionic conductivity compared to conventional liquid electrolytes. Additionally, pre‐impregnating separators with electrolyte before integration into epoxy laminates presents challenges, such as unintended resin‐electrolyte mixing, curing temperature constraints, and electrolyte evaporation during vacuum‐assisted processing. To address these issues, recent research^[^ ^8^ ^]^ has explored methods for encapsulating electrolytes using low oxygen/moisture permeability materials such as PP for lateral shielding while incorporating metal sheets for top and bottom sealing. Moreover, a post‐manufacturing electrolyte injection method has been proposed, allowing for high‐temperature composite curing without electrolyte degradation.^[^ ^7^ ^]^

PP was chosen as the dominant polymeric encapsulant in multifunctional battery composites, owing to its excellent resistance to moisture and oxygen permeation, thermal dimensional stability during lamination, and sufficient interfacial compatibility with reinforcement fibers. Inspired by pouch battery cell structures, our studies^[^ ^8^, ^12^ ^]^ have confirmed that PP effectively prevents electrolyte permeation, ensuring long‐term electrochemical stability. However, PP exhibits insufficient mechanical properties for use as a composite matrix, necessitating an alternative polymer for structural bonding.

To address this challenge, Elium resin was utilized as a thermoplastic polymer matrix, allowing selective PP integration around the electrode regions while enhancing the overall mechanical properties of the structural battery. The manufacturing methodology employed leveraged Elium's ability to be processed in liquid form before polymerization, enabling the fabrication of CFRP prepregs with complex geometries. By using a masking technique, Elium resin was selectively deposited onto designated areas, leaving the electrode region free of resin infiltration. This method preserved electrode integrity while providing precise control over PP bonding adjacent to the electrodes.

Elium, a polymethyl methacrylate (PMMA)‐based thermoplastic polymer, was selected for its mechanical performance, which is comparable to that of traditional epoxy resins, while also offering additional advantages in terms of post‐processing capabilities, scalability for mass production, and recyclability.^[^ ^13^ ^]^ Unlike conventional thermosetting resins, Elium can be applied in its liquid state, allowing for uniform coating on fiber‐reinforced structures and subsequently curing into a solid phase without resin migration during the pressing and curing processes. These characteristics are crucial in preventing resin infiltration into the electrode and electrolyte contamination caused by the resin, which are common issues in the fabrication of thermoset‐based structural batteries. By integrating Elium resin as a structural matrix while selectively incorporating PP in key regions, this study effectively combined mechanical performance, electrolyte stability, and manufacturing flexibility to develop an advanced structural battery composite.

One of the key challenges in developing structural batteries lies in the insufficient bonding strength at the interfaces among current collectors, electrolyte components, and separator layers.^[^ ^1^ ^]^ Discontinuous interfaces in laminated structural batteries contribute to delamination under mechanical loading, significantly compromising durability. This issue becomes more pronounced as the electrode area increases, with mechanical loading further exacerbating interlayer separation, leading to a substantial decline in electrochemical performance.^[^ ^4^ ^]^ Delamination increases the electrode gap and weakens the interfacial bonding between the electrodes and the electrolyte, resulting in higher resistance and a subsequent reduction in charge transport efficiency. To address these challenges, many studies^[^ ^3^, ^4^, ^5^, ^6^, ^7^, ^8^ ^]^ have explored the use of rigid composite face skins to suppress electrode delamination. However, achieving a scalable and structurally robust solution remains a critical challenge.

Compared to conventional 2D laminated structures, 3D stitched architectures offer an efficient method for improving interlaminar properties in a single step by introducing stitching fibers through the thickness direction.^[^ ^14^ ^]^ This reinforcement mechanism enhances delamination resistance and structural integrity, making it particularly beneficial for structural battery applications. Additionally, stitched structures provide a lightweight and effective approach to enhancing interlayer adhesion, as they rely exclusively on fibers for reinforcement.

Among stitching parameters, stitching density constitutes a key variable that determines the balance between reinforcement efficacy and the risk of material damage. Excessive stitching density induces resin‐rich zones and fiber misalignment, whereas a density that is too low may fail to effectively suppress delamination.^[^ ^14^ ^]^ Aramid fibers are frequently selected as a stitching medium due to their high tensile strength, excellent thermal stability, and chemical resistance, providing reliable reinforcement.

In this study, a stitching reinforcement strategy was implemented to minimize the formation of electrode gaps between the anode and cathode, thereby optimizing ion transport pathways. A reinforcement technique employing aramid threads with excellent tensile performance was applied across the stacked configuration comprising the anode collector, separator membrane, and cathode layer. Within this configuration, the influence of three distinct stitching densities on mechanical and electrochemical performance was investigated, and the results were compared and analyzed against unstitched specimens. This strategy preserved minimal separation between electrodes, promoting consistent ionic mobility and improving the overall electrochemical cycling performance. Moreover, under mechanical loading, the stitching structure significantly improved capacity retention by constraining interlayer displacement, thereby mitigating electrochemical performance degradation.

While most studies on structural batteries have focused on single‐cell systems,^[^ ^1^, ^2^ ^]^ practical applications such as electric vehicles and high‐performance drones (e.g., DJI Matrice 350 RTK) demand operating voltages exceeding 40 V or higher. Achieving such voltage levels requires the integration of multiple cells, typically through series connections.

In conventional CFRP‐based laminated structural batteries, series integration is commonly implemented via side‐by‐side configurations. However, this layout suffers from several critical drawbacks: increased electrical losses due to extended interconnect lengths;^[^ ^10^ ^]^ inefficient volumetric utilization caused by inter‐cell dead space;^[^ ^9^ ^]^ degraded structural integrity from physical discontinuities;^[^ ^15^ ^]^ and increased fabrication complexity and non‐uniform current distribution, both of which reduce system‐level reliability and performance.^[^ ^9^, ^10^ ^]^

To overcome these limitations, we propose a vertically stacked structural battery architecture in which multiple unit cells are laminated in series along the thickness direction. This vertical stacking configuration significantly shortens interconnect lengths, thereby reducing ohmic losses, and minimizes inter‐cell spacing, enhancing both spatial and mechanical efficiency. Importantly, the integrated laminate structure maintains in‐plane stiffness while providing uniform electrochemical connectivity and balanced current distribution across cells.

**Figure 1:**
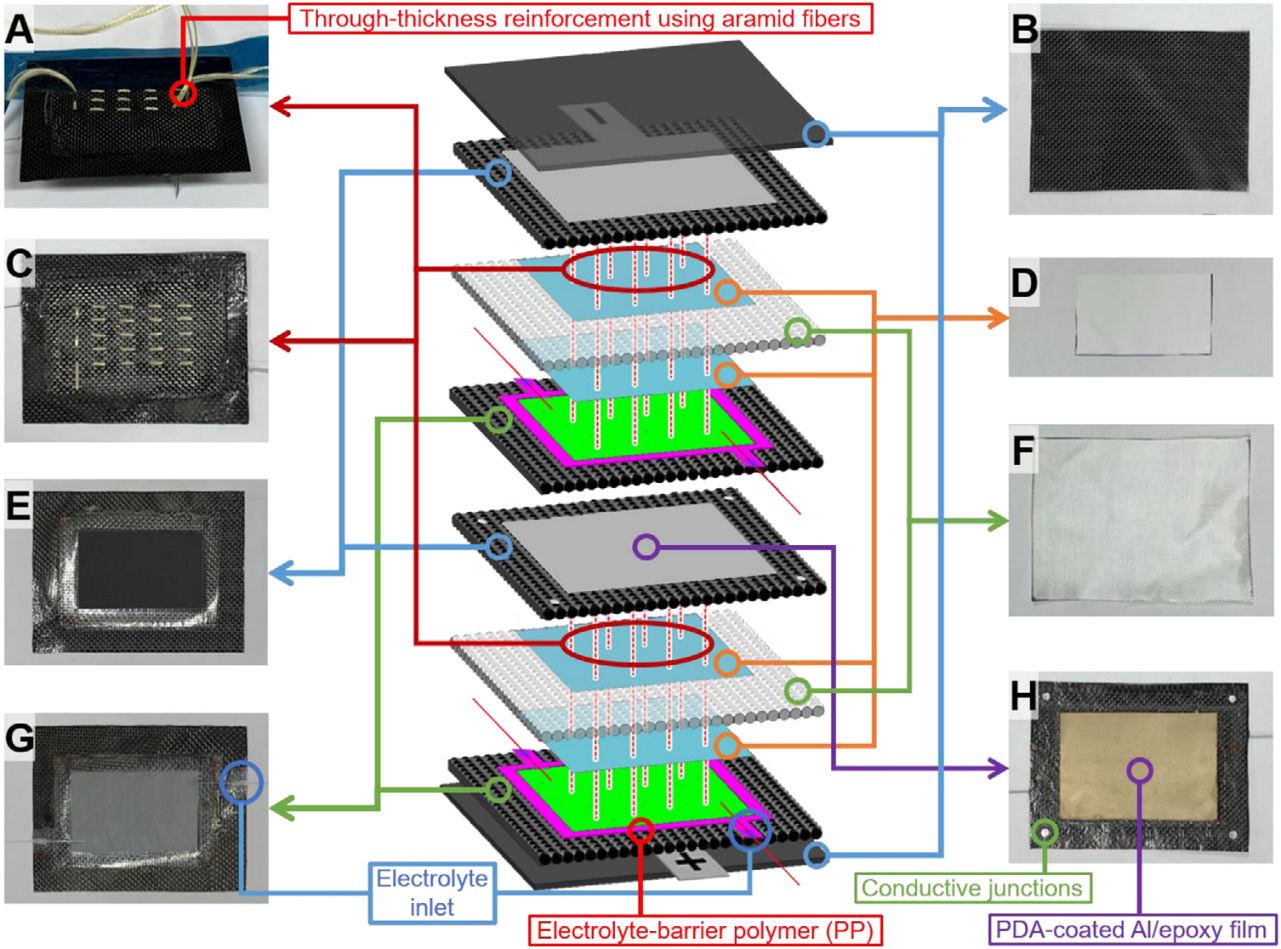
Architecture of a high‐voltage stacked structural battery laminate reinforced by Kevlar through‐thickness stitching. A) Interlayer stitching. B) Outer CFRP face sheet. C) Stitch pattern. D) PP/PE/PP separator. E) LTO electrode on a CFRP current collector. F) Glass‐fabric interleaf (electrical insulation). G) LFP electrode on a CFRP current collector. H) CFRP/Al conductive junctions for series coupling of sub‐cells.

An overview of the proposed stitched structural battery architecture is presented in **Figure** **1**, highlighting key design features such as vertical cell stacking, through‐thickness aramid stitching for electrode alignment, selective integration of PP and Elium polymers, and the post‐injection pathway for liquid electrolyte. Beyond fiber‐reinforced structural batteries, the findings of this study have broader implications for pouch cell battery technology, where maintaining electrode alignment and electrolyte stability is equally critical. Combining through‐thickness reinforcement patterns with thermoplastic matrix systems presents a versatile and scalable fabrication route, with the potential to transform energy storage technologies in fields ranging from aerospace platforms and e‐mobility solutions to autonomous devices and advanced consumer electronics.

## 2 Experimental Section

### 2.1 Fabrication and Characterization of CFRP Current Collector

**Figure 2:**
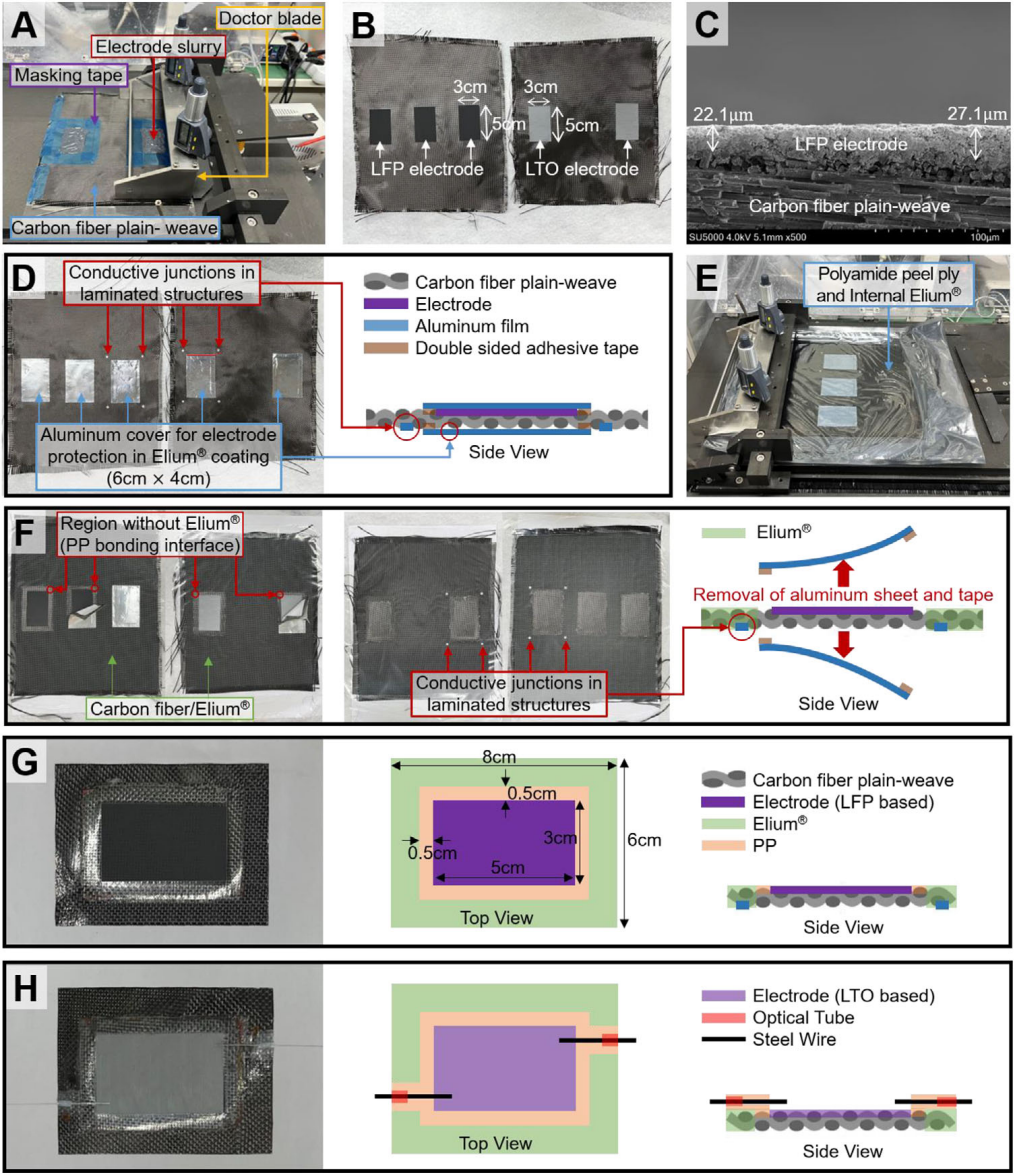
Fabrication sequence of a CFRP‐based current collector. A) Doctor‐blade‐assisted slurry coating on carbon fiber fabric. B) Vacuum drying to form LFP and LTO electrode layers. C) Cross‐sectional SEM of the electrode‐coated composite. D) An aluminum sheet applied to protect the electrodes during resin processing. E) Uniform coating of Elium resin to form prepreg. F) Prepreg finalized by curing and removal of the protective aluminum. G) LFP‐integrated carbon/Elium collector with PP bonding zones. H) LTO‐integrated collector with electrolyte inlet for cell assembly.

The CFRP current collector was fabricated through a sequence of steps designed to precisely define the electrode region and enable integration within a structural battery configuration, as shown in **Figure** **2**.

For the cathode, a slurry was formulated with 78 wt.% LiFePO_4_ (LFP), 10 wt.% carbon black (CB), and 12 wt.% polyvinylidene fluoride (PVDF), while the anode used 82 wt.% Li_4_Ti_5_O_12_ (LTO), 8 wt.% CB, and 10 wt.% PVDF. Each 4 g portion of electrode mixture was blended with 11 mL of 1‐methyl‐2‐pyrrolidone (NMP) using a high‐speed centrifugal mixer operating under vacuum conditions, aiming to suppress bubble formation and achieve homogeneous dispersion.

To localize electrode coating to a designated 3 cm × 5 cm area on the carbon fabric, a masking method using Teflon tape was employed directly on the plain‐weave carbon fiber substrate (Figure 2A). The prepared slurry was then cast within this masked region using a doctor blade set to a 200 µm gap between the blade and substrate, ensuring uniform electrode thickness across the exposed area (Figure 2A). After deposition, the electrodes were vacuum‐dried (solvent removal and PVDF binder consolidation) using a ramp of 0.5 °C min^−1^ to 100 °C and a 3 h hold. Subsequently, the Teflon tape used to mask the electrode region during slurry casting was carefully removed (Figure 2B).

In order to assess the consistency of the electrode thickness and its interfacial conformity with the carbon fiber substrate, the coated region was sectioned perpendicular to the surface and examined using scanning electron microscopy (SEM). The SEM images (Figure 2C) confirmed that the electrode layer was uniformly coated with a thickness of ≈22–28 µm, indicating precise and consistent deposition on the carbon fiber substrate.

A rectangular masking frame, extending 5 mm beyond the 3 cm × 5 cm electrode area, was applied using acrylic double‐sided tape. A 0.1 mm‐thick aluminum sheet, cut to 4 cm × 6 cm, was then placed over this masked region. Importantly, the aluminum sheet was not in direct contact with the electrode surface; instead, it was attached exclusively along the outer masked boundary using acrylic‐based double‐sided adhesive tape (Figure 2D). This area was intentionally reserved to facilitate PP attachment during subsequent processing. The structural rationale stems from the inferior resistance of Elium, a PMMA‐based polymer, to moisture and oxygen ingress when compared with polypropylene.^[^ ^11^ ^]^ Therefore, PP was integrated around the electrode region instead of Elium, ensuring that PP directly interfaced with the electrolyte. Furthermore, a 5 mm‐wide PP barrier layer was adopted by referencing the standard pouch battery cell structure. Preliminary experimental studies confirmed that PP provides superior electrolyte retention performance compared to Elium, further validating the necessity of PP integration for long‐term electrochemical stability.

One face of a 3‐mm‐diameter, 0.1‐mm‐thick aluminum disc was coated with silver paste and then thermally bonded under pressure to the edge of the CFRP electrode (Figure 2D). These discs correspond to the four small circular “conductive junctions” highlighted in Figure 1H. They were incorporated solely at the interlaminar mid‐plane to implement series interconnections between the vertically stacked cells.

Preparation of the CFRP prepreg involved coating liquid thermoplastic resin Elium 150, pre‐mixed with 2 wt.% Perkadox CH‐50X initiator using the same planetary mixer (1500 RPM, 1 min), onto both sides of the carbon substrate placed within PA/PO peel ply (Figure 2E). Coating thickness was controlled via a doctor blade set at 400 µm. After curing at ambient conditions (≈10 h), the peel ply and aluminum masking layers were removed, yielding a CFRP prepreg ≈300 µm thick (Figure 2F).

The CFRP laminate obtained from the preceding steps was trimmed to a final dimension of 8 cm × 6 cm in accordance with the structural battery module design specifications. PP films of 5 mm width and 0.1 mm thickness were pre‐bonded to the electrode perimeter using a soldering iron (Figure 2G).

Furthermore, to facilitate electrolyte injection and allow trapped air to escape, two injection ports were integrated into the design. In the CFRP prepared with the LTO electrode, additional PP layers were affixed around the electrolyte inlet and outlet points. A PMMA‐based optical fiber tube (outer diameter: 0.62 mm, inner diameter: 0.38 mm) was cut into 5 mm segments, into which a steel wire with a diameter of 0.35 mm was placed. The steel wire was coated with a fluoropolymer release agent and dried for 12  h before use. The release agent facilitated easy removal of the steel wire during the electrolyte injection step, while the fluoropolymer's excellent chemical stability ensured minimal reactivity with the electrolyte.

This component was pre‐bonded with heat to create embedded electrolyte injection channels (Figure 2H). Upon later removal of the steel wire, a PP‐encased path remained, maintaining chemical integrity and barrier functionality during electrolyte infusion. This multilayered CFRP structure with defined electrodes, electrical junctions, and integrated electrolyte barrier regions constituted a foundational element for the stitched structural battery system.

### 2.2 Assembly of Single‐Cell Structural Battery with Stitched Structures

**Figure 3:**
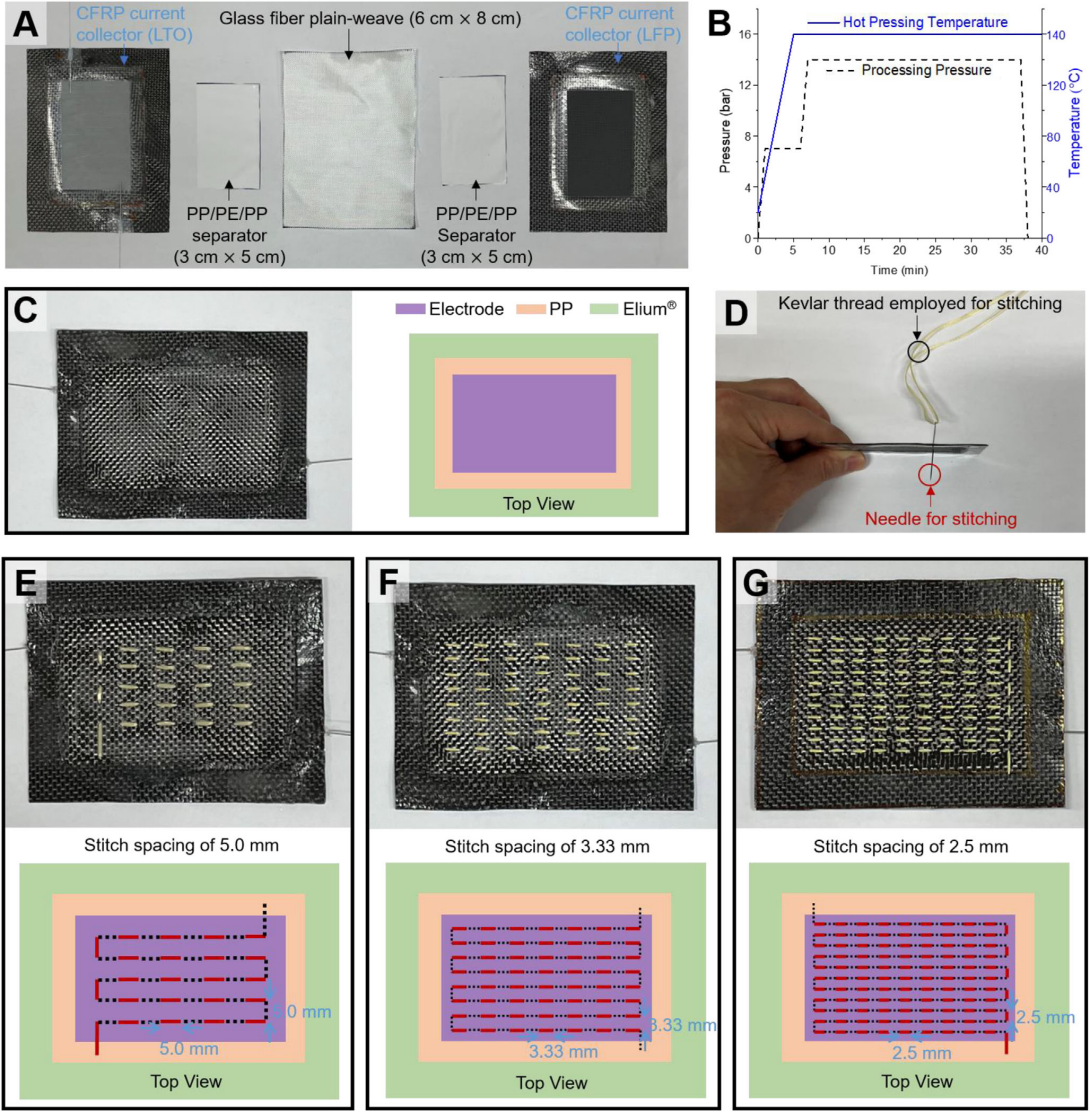
Lamination and stitching process. A) Assembly of laminated components. B) Pressure–temperature cycle for hot‐pressing. C) Laminated structure after hot‐pressing consolidation. D) Side view of stitching. Top views of stitched structural batteries with varying stitch spacings: 5.0 mm (E), 3.33 mm (F), and 2.5 mm (G).

**Figure** **3** presents the fabrication sequence for a stitched single‐cell structural battery. As shown in Figure 3A, the structural assembly consisted of multiple functional layers arranged in a specific order: the LTO‐based CFRP current collector (6 cm × 8 cm) was placed at the bottom, followed sequentially by a 3 cm × 5 cm PP/PE/PP separator, a glass fiber plain‐weave sheet (6 cm × 8 cm), another identical separator, and finally the LFP‐based CFRP current collector (6 cm × 8 cm) at the top.

The intermediate glass fiber plain‐weave served a dual purpose: it provided mechanical integrity to the laminated system and electrically isolated the anode and cathode layers. To further ensure robust insulation between electrodes during the high‐pressure curing process, thin (16 µm) multilayer PP/PE/PP membranes (Celgard QT17P2HX) were inserted between each CFRP electrode and the glass fiber sheet. These separators, trimmed to precisely match the 3 cm × 5 cm electrode area, were not bonded chemically to either Elium resin or PP films but were simply embedded between the layers. This placement strategy ensured enhanced bonding between the resin and the adjacent glass fiber, while the separator maintained its structural form without distortion, even under hot‐pressing conditions at 140 °C.

Figure 3B shows the thermal‐compression profile employed to unify the laminate structure. A temperature of 140 °C and pressure of 14 bar were applied and sustained for a minimum of 30 min to facilitate close interfacial contact and effective bonding of the Elium matrix. After consolidation, the layered architecture is shown in Figure 3C (side view), confirming the intended stacking sequence. To maintain electrical isolation between the electrodes after assembly, the choice of stitching fiber was critical. A high‐strength aramid yarn (Kevlar KM2) was selected due to its non‐conductive nature and stability under elevated temperature and pressure. As shown in Figure 3D, a stitching needle of 63 µm diameter was used to introduce the Kevlar fiber (≈45 µm in bundle diameter) through the laminate stack. The stitching was confined to the active electrode region within the PP boundary, and the Kevlar yarn was tensioned during stitching to ensure firm contact and reduced slack. The tensioned pattern also contributed to better structural integrity upon curing.

This study compared the electrochemical and mechanical performance of four structural battery configurations distinguished by stitching density: a non‐stitched structure (Figure 3C; non‐stitched SB), a sparsely stitched configuration with 5 mm intervals (Figure 3E; Loosely stitched SB), a medium‐density pattern with 3.33 mm intervals (Figure 3F; moderately stitched SB), and a high‐density arrangement with 2.5 mm intervals (Figure 3G; densely stitched SB). The effects of these different stitching strategies on interfacial bonding, mechanical reinforcement, and electrochemical isolation were systematically investigated.

### 2.3 Fabrication of High‐Voltage Structural Battery via Multi‐Cell Lamination

**Figure 4:**
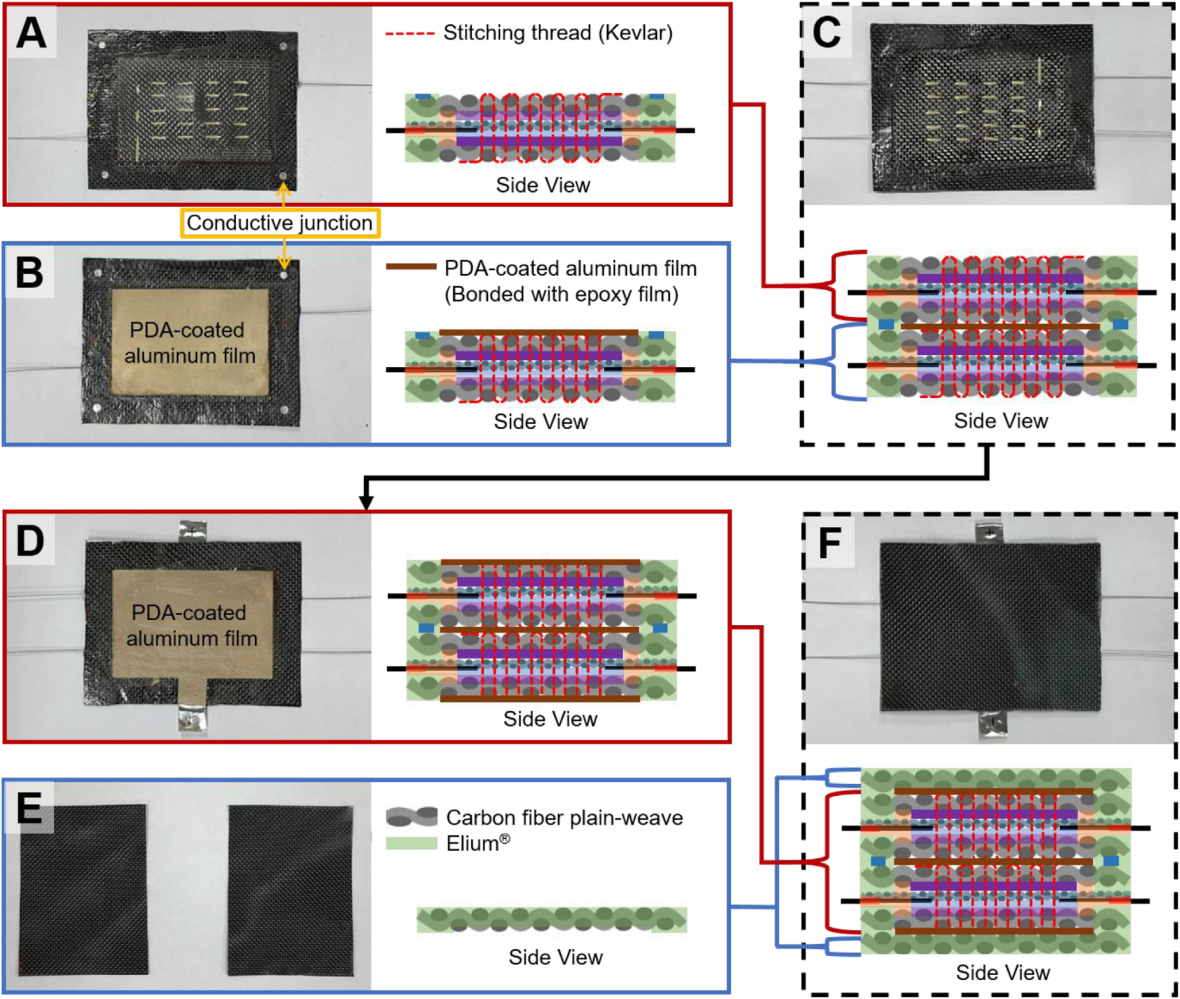
Assembly process of the aluminum barrier and the high‐voltage laminated structure. A) Single‐layer structural battery without an aluminum barrier. B) Single‐layer structural battery integrated with an aluminum film. C) Two‐cell stacked configuration formed by laminating (A,B). D) Integration of aluminum film serving as both a current connector and a barrier layer. E) External carbon/Elium composite layers. F) Final configuration of the multilayer structural battery.

**Figure** **4** illustrates the sequential steps involved in assembling a high‐voltage structural battery by laminating two cells previously fabricated as depicted in Figure 4A,B. In structural battery systems, achieving complete electrolyte encapsulation was critical for sustained performance. While the peripheral PP barrier effectively prevented electrolyte migration laterally, complete electrolyte barrier capability in the face direction with Elium alone was insufficient. Metals, benefiting from their densely ordered atomic structures, exhibited superior barrier properties even at significantly reduced thicknesses. Aluminum was particularly attractive among metallic materials due to its advantageous strength‐to‐weight ratio. Thus, to effectively block electrolyte permeation between adjacent cells during lamination, a thin aluminum film (6 µm, A‐ALFo ACEY) was attached to the Elium‐free region (4 cm × 6 cm), as depicted in Figure 4B. This aluminum film was bonded to the underlying structure using a thin epoxy film adhesive (Redux 312, thickness: 0.05 mm).

The previous experimental outcomes^[^ ^8^, ^16^ ^]^ indicated insufficient interfacial adhesion between the aluminum film and epoxy adhesive, leading to compromised mechanical properties. Therefore, in this study, dopamine functionalization of the aluminum film surface was adopted to enhance its adhesion properties. The dopamine coating process was consistent with methods previously established in the literature:^[^ ^17^ ^]^ Dopamine hydrochloride (2 g L^−1^) and tris‐base (≈1.2 g L^−1^ for pH adjustment to 8.5) were dissolved in deionized water; the aluminum films were immersed in this solution at room temperature under magnetic stirring (600 rpm) for 24 h, subsequently rinsed three times with deionized water, and vacuum dried at 50 °C for 6 h. A single cell with the dopamine‐treated aluminum film attached is presented in Figure 4B.

To assemble a high‐voltage laminated battery, an additional epoxy adhesive film layer was applied on the dopamine‐treated aluminum film surface. During the hot‐press lamination step, conductive junctions positioned at opposing corners of each cell were precisely aligned and bonded under heat and pressure, ensuring both electrical interconnection and mechanical integrity between the stacked cells. The resulting high‐voltage configuration comprising two laminated cells is shown in Figure 4C.

For improved barrier properties against moisture and oxygen ingress, aluminum films were similarly bonded to the top and bottom surfaces of the laminated high‐voltage cell structure. In this stage, regions intended for electrical contact were selectively masked using Teflon tape to prevent dopamine coating; consequently, these uncoated areas maintained direct electrical contact with the CFRP electrode surfaces. Moreover, the area of the epoxy adhesive film was intentionally reduced by ≈1 mm relative to the aluminum film dimensions, creating an unbonded peripheral zone. This design allowed direct contact between the aluminum film edges and the CFRP current collector during the hot‐press consolidation step. Thus, this direct contact facilitated an efficient electrical conduction pathway, enabling the aluminum film to act simultaneously as an effective moisture and oxygen barrier and as an auxiliary current collector connected to the CFRP electrode.

In this laminate, the CFRP served as the current collector for both the LTO and LFP electrodes (Figure 1E,G). For electrical routing and series connection, an aluminum T‐shaped terminal was bonded to the opposite face of each CFRP collector; the aluminum film simultaneously acted as a moisture/oxygen barrier and an auxiliary current collector through direct contact with the CFRP, as shown in Figure 4D. To enhance mechanical robustness at the exposed terminal areas, a 0.2‐mm aluminum reinforcement sheet was attached.

Conventional Li‐ion cells typically employed aluminum for the positive and copper for the negative current collectors; however, in this structural battery used aluminum was deliberately used for both electrodes to reduce mass and to enable reliable bonding to the epoxy matrix. Adhesion between aluminum and the epoxy adhesive was further improved via dopamine functionalization of the aluminum surface, following established procedures.

To safeguard the bonded aluminum surfaces and enhance structural integrity, Carbon/Elium prepregs were fabricated following the aforementioned process and subsequently trimmed into 6 cm × 8 cm sheets (Figure 4E). These prepregs functioned as protective skins for the structural battery, providing external mechanical protection and additional sealing against external environmental influences. These prepreg sheets were applied to both the upper and lower surfaces of the structural battery through hot‐press lamination, employing epoxy adhesive films to bond the protective skins firmly onto the aluminum films.

As shown in Figure 4F, the finalized high‐voltage structural battery adopted a multi‐layer configuration that integrated through‐thickness stitching and dopamine‐modified aluminum layers to improve mechanical robustness.

### 2.4 Electrolyte Injection and Sealing Process for Structural Battery Activation

**Figure 5:**
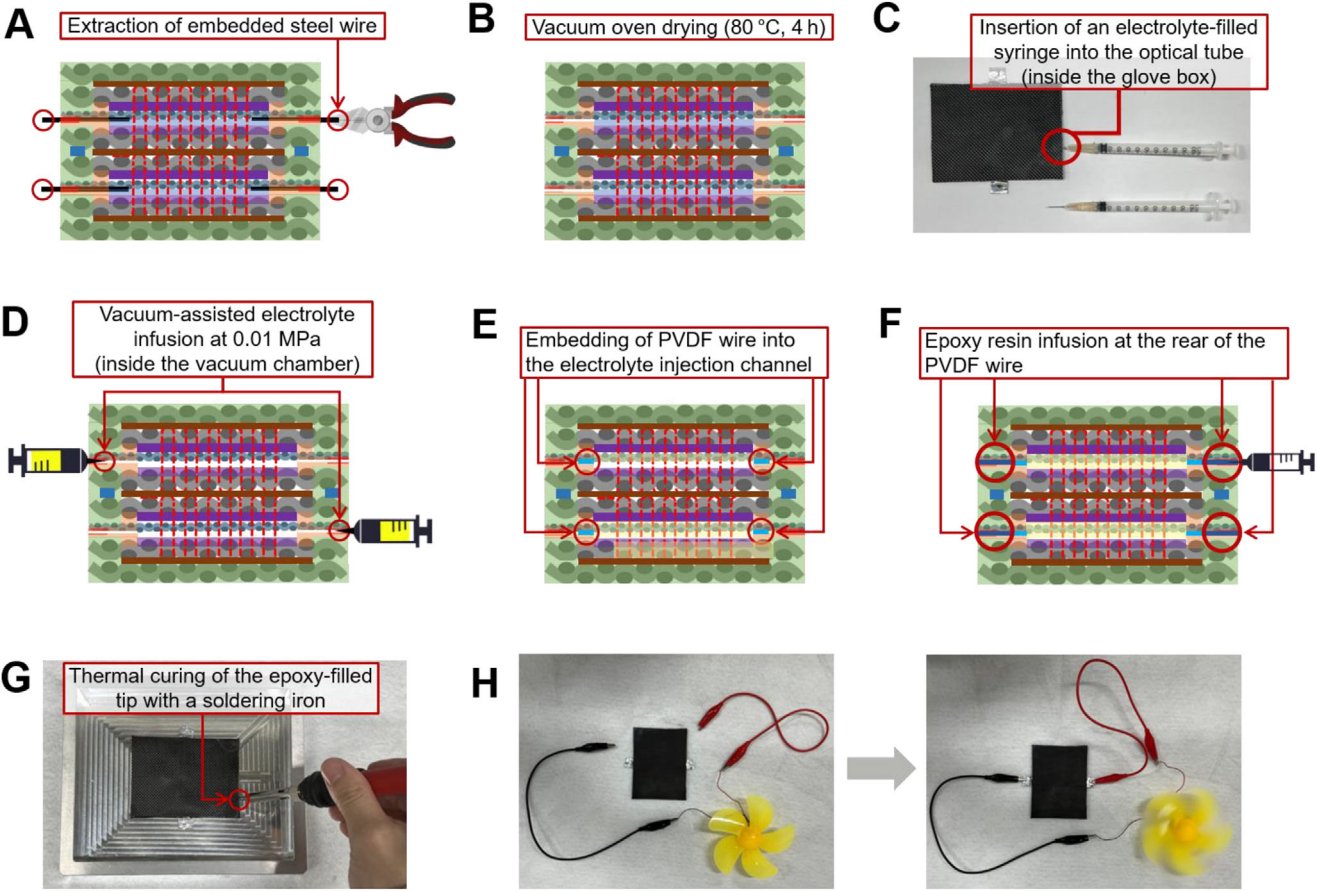
Electrolyte injection process. A) Removal of embedded steel wire to create an electrolyte injection channel. B) Structural battery after steel wire removal. C) Connection of a syringe to the optical tube inlet on the structural battery's side. D) Vacuum‐assisted electrolyte infusion into the internal structure. E) Insertion of a PVDF wire into the electrolyte injection channel for sealing. F) Backfilling of epoxy resin at the rear of the PVDF wire to ensure encapsulation. G) Thermal curing of the epoxy‐sealed tip using a soldering iron to finalize the sealing process. H) Demonstration of structural battery operation.

**Figure** **5** illustrates the sequential process used to remove the internal guide wire, inject electrolyte, and complete sealing of the high‐voltage structural battery. To begin the electrolyte port activation process, localized heating was applied at the interface between the optical tube and the embedded steel wire using a hot air blower set to ≈130 °C (Figure 5A). This thermal input softened the surrounding polymer and reduced interfacial resistance, enabling safe extraction of the internal steel wire without damaging the surrounding structure. After wire removal, the battery was placed inside a vacuum oven at 80 °C for ≈4 h to facilitate the evaporation of residual solvents and volatile compounds (Figure 5B).

Subsequently, the dried structural battery was transferred into an argon‐filled glove box to maintain a moisture‐free environment during electrolyte infiltration. All electrolyte injection procedures were conducted inside a nitrogen‐filled glove box to prevent contamination. A syringe needle with a diameter of 0.36 mm was selected for electrolyte injection, as it closely matched the diameter of the optical tube hole left after steel wire removal, ensuring a precise and secure fit. Following electrolyte filling, the syringe needle was inserted into the optical tube hole on the side of the structural battery (Figure 5C). The battery was then transferred to the transition chamber of the glove box, and the chamber pressure was reduced to 0.01 MPa. In this process, as the air inside the structural battery was rapidly evacuated in a vacuum environment, the liquid electrolyte was drawn into the structural battery (Figure 5D). This procedure was repeated multiple times, and the process continued until the residual liquid electrolyte in the syringe no longer significantly decreased even after maintaining the vacuum state in the transition chamber for approximately one minute.

To seal the injection ports, a 0.34 mm‐diameter, 8 mm‐long polyvinylidene fluoride (PVDF) wire (KOVERT CLASSIC) was inserted into each optical tube, occupying the void left by the previously removed steel wire (Figure 5E). Upon insertion, the battery was taken out of the electrolyte bath, and a syringe fitted with a 0.36 mm‐diameter needle was used to inject a pre‐mixed epoxy resin into the remaining void of the optical tube, effectively encapsulating the PVDF wire and sealing the electrolyte channel (Figure 5F). Final curing of the epoxy was performed using a soldering iron, applying localized heat to the tip of the epoxy‐filled port to initiate thermal polymerization and ensure a complete seal (Figure 5G). The fully sealed and activated structural battery successfully achieved a high‐voltage output of 5 V and powered an external load, as shown in Video S1 (Supporting Information) and Figure 5H.

## 3 Results and Discussion

### 3.1 SEM and EDS Analysis of Stitch‐Penetrated Regions

To investigate the material transfer and damage induced by through‐thickness stitching, SEM and energy dispersive X‐ray spectroscopy (EDS) analyses were conducted on the laminated components—specifically focusing on the CFRP current collectors (coated with either LFP or LTO composite slurry), PP/PE/PP separators, and glass fiber plain‐weave fabric. To minimize structural and electrochemical damage, a needle with a diameter of 63 µm was selected, which was the smallest possible size capable of accommodating aramid fiber bundles (≈45 µm in diameter). All samples analyzed were stitched using the same procedure as in the actual structural battery fabrication process.

**Figure 6:**
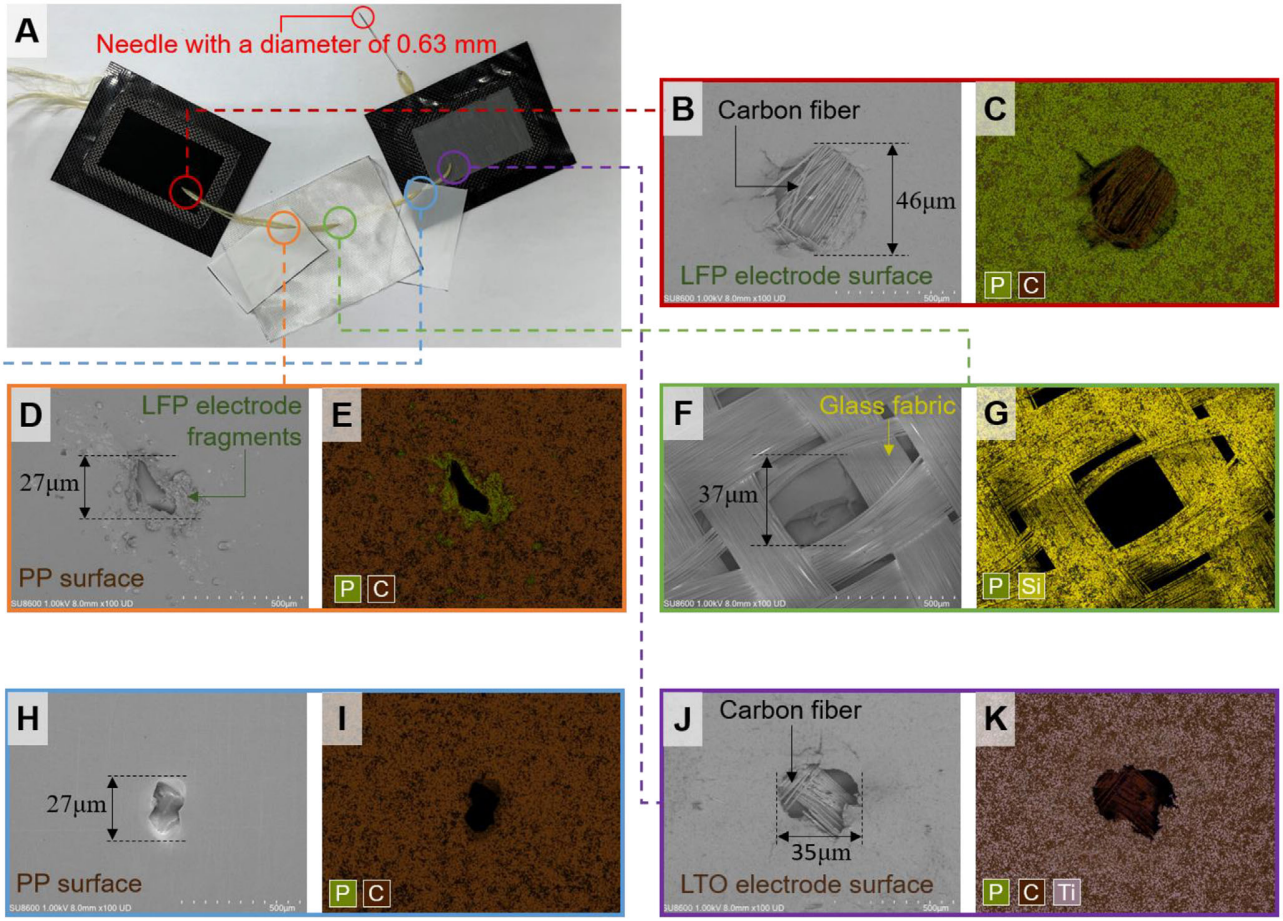
SEM and EDS analyses of stitch‐penetrated regions in laminated components. A) Stitched configuration of the laminated components. SEM image (B) and EDS mapping (C) of the LFP‐electrode CFRP. SEM image (D) and EDS mapping (E) of the PP/PE/PP separator located beneath the LFP‐electrode CFRP. SEM image (F) and EDS mapping (G) of the glass fabric separator. SEM image (H) and EDS mapping (I) of the PP/PE/PP separator located beneath the glass fabric layer. SEM image (J) and EDS mapping (K) of the LTO‐electrode CFRP.

As illustrated in **Figure** **6**A, the stitch sequentially penetrated the LFP‐coated CFRP current collector, PP/PE/PP separator, glass fiber fabric, another PP/PE/PP separator, and finally the LTO‐coated CFRP current collector. SEM analysis of the LFP electrode surface (Figure 6B) revealed that the coating was partially removed near the penetration site, exposing underlying carbon fibers. EDS mapping (Figure 6C) confirmed localized loss of phosphorus (P), a signature element of the LFP active material, indicating that conductive electrode fragments had been displaced by the stitching process. The diameter of this localized damage zone was ≈46 µm.

In the subsequent PP/PE/PP separator layer, SEM (Figure 6D) and EDS analyses (Figure 6E) revealed the presence of P and C elements surrounding the perforation. These are detached LFP fragments originating from the damaged electrode layer above. Notably, the separator's elastic properties limited the perforation diameter to ≈27 µm—substantially smaller than that of the CFRP electrode layer.

In contrast, the EDS mapping of the next layer, the glass fiber fabric (Figure 6F,G), showed no significant P content, indicating that LFP fragments did not further propagate beyond the first separator layer. Likewise, no electrode‐derived elements were detected in the second PP/PE/PP separator (Figure 6H,I) or the LTO‐coated CFRP electrode layer (Figure 6J,K). These findings strongly support that the multilayered configuration of PP/PE/PP/glass fabric/PP/PE/PP acts as an effective physical barrier, preventing conductive particle migration and potential internal short circuits.

Such mitigation is critical, as conductive debris—particularly micro‐sized carbon fibers or LFP/LTO particles—could bridge the cathode and anode current collectors, leading to electrical shorting. Indeed, control experiments confirmed that when only glass fiber was used as the separator, stitching‐induced carbon fiber fragments occasionally connected the electrodes, causing electrical failure. In contrast, when PP/PE/PP separators were placed on both sides of the glass fabric, the configuration reliably prevented such shorts. Electrical testing across multiple stitched samples confirmed this insulating effect.

Moreover, although PP/PE/PP separators offer excellent electrical insulation and effectively block electrode debris, their inherently low interfacial bonding strength can compromise the overall laminate integrity. Therefore, in laminate architectures where compatibility with Elium resin is critical, replacing the PP/PE/PP separator with a single layer of glass fabric can substantially enhance interlaminar adhesion. Based on this rationale, a hybrid configuration is proposed: the glass fabric layer spans the full area of the laminate to provide structural cohesion, while the PP/PE/PP separator is selectively applied only to the localized electrode regions. This approach ensures that the PP/PE/PP layer does not bear structural load, allowing the glass fabric to act as the primary load‐bearing interleaf, thus preserving both mechanical robustness and electrochemical isolation in the structural battery.

The penetration diameters measured in CFRP current collectors were ≈37–46 µm, while those in PP/PE/PP separators were significantly smaller (≈27 µm), attributed to their higher compliance. This dimensional disparity highlights the mechanical filtering function of the PP/PE/PP layer, acting as a buffer that physically restrains electrode fragments and carbon fiber particles from reaching the opposing electrode. In summary, the multilayered separator configuration of PP/PE/PP/glass fabric/PP/PE/PP has proven to be highly effective in preventing stitching‐induced internal short circuits in structural battery composites.

**Figure 7:**
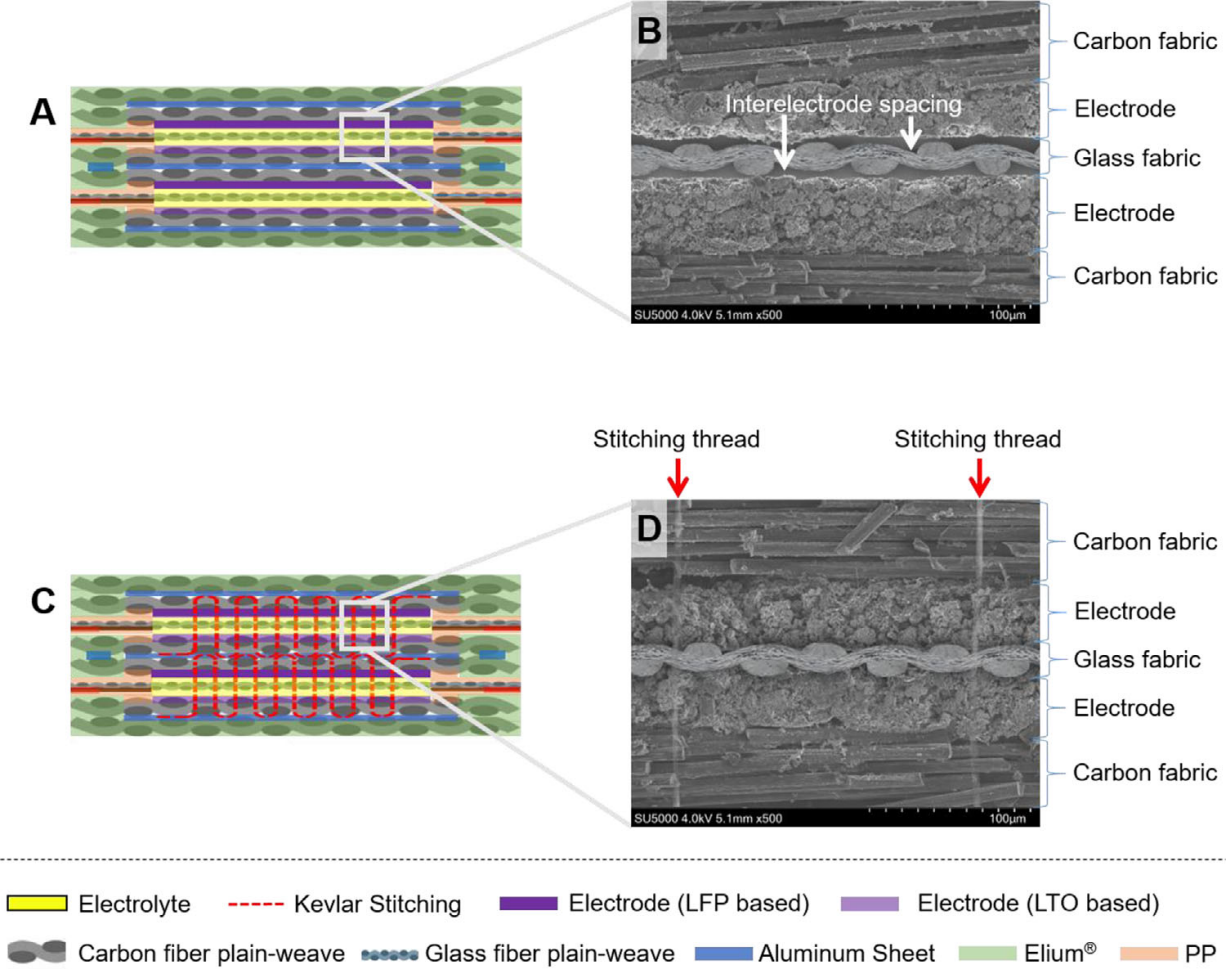
Cross‐sectional SEM analysis of the structural battery laminate. A) Cross‐sectional schematic of the unstitched laminate and B) the corresponding SEM. C) Cross‐sectional schematic of the laminate reinforced with through‐thickness stitching and D) the corresponding SEM.

**Figure** **7** compares cross sections of devices fabricated identically to those in the Experimental Section. In the unstitched laminate (Figure 7B), the glass‐fabric separator exhibits local waviness and intermittent debonded pockets against both electrodes, resulting in enlarged interelectrode spacing at the crests and troughs. In contrast, the stitched laminate (Figure 7D) shows two through‐thickness aramid threads traversing the electrode–separator stack and locally compacting the plies.

Stitched laminates consistently maintained interelectrode spacing comparable to the diameter of the separator filaments—near contact with only micrometer‐scale, electrolyte‐filled residual voids—whereas unstitched laminates exhibited gaps of several micrometers caused by separator undulation and local debonding. Maintaining a small interelectrode spacing is essential for efficient ionic transport and capacity retention. Conversely, weak interlayer bonding increases the interelectrode spacing and degrades energy‐storage performance, a limitation commonly reported for laminated structural batteries that lack through‐thickness reinforcement.

### 3.2 Microstructural Characterization of Stitched Electrode‐Coated CFRP Current Collectors

**Figure 8:**
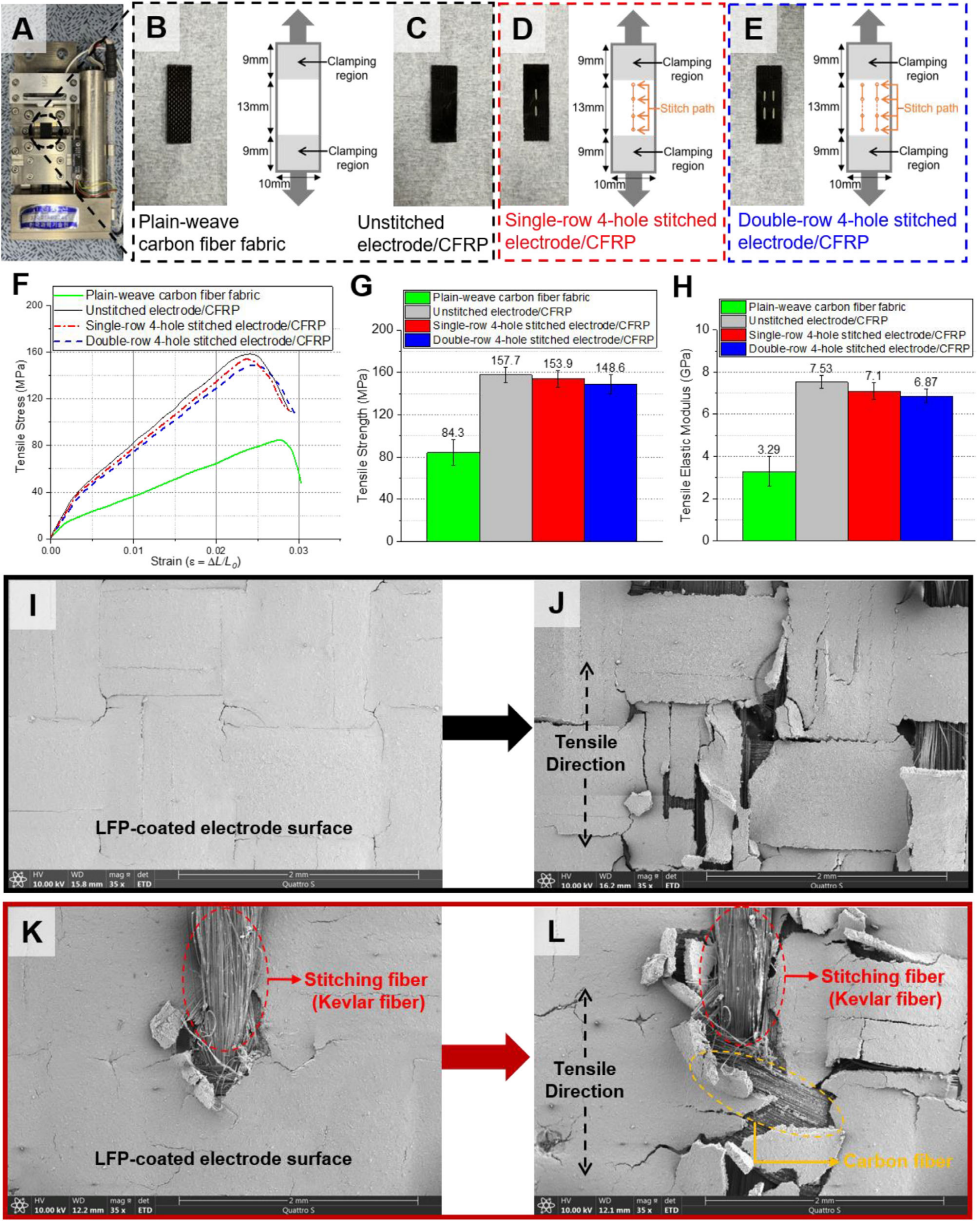
Microstructural and mechanical evaluation of electrode/CFRP specimens. A) Micro‐tensile testing setup. B) Plain‐weave carbon‐fiber fabric specimen. Electrode/CFRP specimens: unstitched (C), single‐row four‐hole stitched (D), and double‐row four‐hole stitched (E). F) Stress–strain curves. G) Tensile strength. (H) Elastic modulus. SEM micrographs of the unstitched sample obtained prior to (H) and following (I) tensile testing. SEM micrographs of the stitched sample obtained prior to (J) and following (K) tensile loading.

To investigate the effect of stitching on the mechanical performance and microstructural integrity of electrode‐coated carbon fiber reinforced polymer (Electrode/CFRP) current collectors, tensile tests and subsequent SEM analyses were performed using a micro‐tensile testing apparatus (**Figure** **8**A). Specimens were fabricated following the same procedure described in the experimental methods, which involved coating a plain‐weave carbon fiber fabric with LFP electrode slurry followed by curing. Each tensile specimen measured 10 mm in width and 31 mm in length, with a gripping area of 9 mm at each end to ensure proper fixation in the tensile apparatus. A pristine plain‐weave carbon‐fiber fabric served as the reference (Figure 8B). Three electrode/CFRP configurations were evaluated: unstitched (Figure 7C), single‐row four‐hole stitched (Figure 8D), and double‐row four‐hole stitched (Figure 8E). Stitch patterns were applied uniformly, as shown in Figure 8D,E.

Tensile tests were conducted at a strain rate of 0.1 mm min^−1^, and representative stress–strain curves for each specimen type are shown in Figure 8F. All three specimen configurations exhibited similar overall stress–strain behavior. Notably, due to the delicate nature of the electrode‐coated surfaces, the gripping pressure was minimized to prevent premature failure at the grip ends, resulting in unavoidable initial slack and non‐linear response at the very early stage of loading. Therefore, elastic modulus values (Figure 8H) were derived from the linear region of the curves, specifically within the strain range of ≈0.005–0.01.

To isolate and quantify the effect of the electrode coating on the mechanical response of our CFRP‐collector structural battery, we first established a baseline with uncoated plain‐weave carbon‐fiber fabric. The baseline showed a tensile strength of 84.3 MPa and a tensile modulus of 3.29 GPa (Figure 8G,H). After applying the LFP‐based electrode to the same fabric (unstitched Electrode/CFRP), the tensile strength increased to 157.7 MPa and the modulus to 7.53 GPa—gains of 87.1% and 128.9%, respectively (Figure 8G,H). These results indicate that the electrode layer is mechanically active rather than a passive add‐on. Mechanistically, the electrode composite (active material, conductive carbon, and polymeric binder) forms a continuous, polymer‐rich matrix that fills inter‐tow voids, enhances interfilament shear transfer, and constrains yarn slippage under load, thereby increasing both stiffness and strength. Taken together, these results show that, within the CFRP‐collector architecture, the electrode coating acts as a thin, load‐bearing matrix that improves interfilament stress transfer and, consequently, increases both stiffness and strength relative to the uncoated carbon‐fiber fabric.

The unstitched Electrode/CFRP specimen exhibited a tensile strength of 157.7 MPa and an elastic modulus of 7.53 GPa (Figure 8C). In contrast, the single‐row four‐hole stitched specimen (Figure 8D) demonstrated a reduction of 5.2% in tensile strength (153.9 MPa) and a 6.5% reduction in elastic modulus (7.1 GPa). Furthermore, the double‐row four‐hole stitched specimen (Figure 8E) showed even greater reductions, with tensile strength decreasing by 9.5% (148.6 MPa) and elastic modulus by 15% (6.87 GPa). This trend indicates that increasing stitch density correlates negatively with mechanical performance. The observed reductions in mechanical performance are largely due to localized fiber breakage and structural disturbances introduced during the stitching procedure.

Post‐test SEM analyses provided insights into damage mechanisms at the microstructural level. For the unstitched specimen, cracks predominantly initiated and propagated around woven carbon fiber intersections, causing progressive matrix and fiber damage (Figure 8H,I). Stitched specimens showed additional localized crack propagation around the stitch sites (Figure 8J,K). Although some degree of localized damage was introduced by stitching, the overall level of electrode deterioration following tensile testing was similar in both stitched and non‐stitched configurations, implying minimal additional impact from the stitching.

Following tensile testing, the stitched fibers retained their position without exhibiting any noticeable signs of slippage or dislodgement. Instead, the woven carbon fiber structure remained intact (Figure 8K), demonstrating that the aramid stitching fibers were effectively restrained under mechanical loading conditions. This observation suggests that stitched electrode‐coated CFRP composites retain structural integrity and stitching effectiveness under moderate tensile loads.

### 3.3 Electrochemical Characterization of Structural Batteries in the Absence of Mechanical Loading

**Figure 9:**
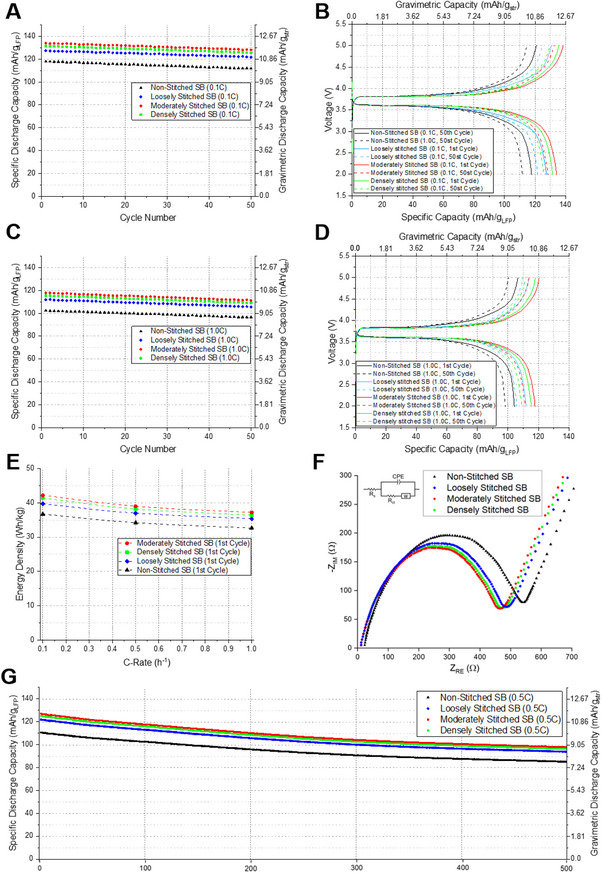
Galvanostatic charge–discharge profiles of structural batteries with varying stitching densities under no applied mechanical load. Specific capacity evolution over 50 cycles at 0.1C (A) and 1C rates (C), respectively. Charge/discharge voltage profiles at 0.1C (B) and 1.0C (D). E) Comparison of energy density. F) EIS characterization. G) Long‐term cycling performance assessed at 0.5C.

Prior to rate/cycling tests, cells were conditioned by three galvanostatic formation cycles at a low current (0.05 C). For the unstitched structural battery (non‐stitched SB), the first‐cycle discharge specific capacity was 123.6 mAh g^−1^ and decreased to 120.2 mAh g^−1^ by the third cycle; the irreversible loss during formation was therefore 3.4 mAh g^−1^ (2.8% relative to the first cycle). Post‐formation, the cells exhibited stable capacities at 0.1–1C, and subsequent voltage‐profile and specific‐capacity data in **Figure** **9** were collected only after completion of the formation protocol.

The electrochemical performance of four structural battery variants was evaluated: non‐stitched SB, loosely stitched SB (stitch spacing of 5 mm), moderately stitched SB (stitch spacing of 3.33 mm), and densely stitched SB (stitch spacing of 2.5 mm). Constant current cycling was performed at various C‐rates (0.1C, 0.5C, and 1C), using the theoretical capacity of the LFP cathode material (170 mAh g^−1^) as the basis.

Figure 9 illustrates the charge–discharge profiles measured under conditions devoid of external mechanical loads. Three metrics were employed to characterize battery capacity. To quantify specific capacity (mAh g_LFP_^−1^), the total discharge was referenced to the corresponding mass of active LFP in the cathode layer. In parallel, the overall gravimetric capacity (mAh g_str_^−1^) was assessed relative to the total structural battery mass, thereby reflecting the system‐level energy contribution per unit weight. The energy density (Wh kg^−1^) was computed using Equation (1), integrating the voltage–capacity curve over the discharge period:

(1) $$\mathrm{Energy}\;\mathrm{density}\;\left(\mathrm{Wh}\;\mathrm{kg}-1\right)=\frac{I}{M}\int_{0}^{t}V\left(t\right)\mathrm{dt}$$

In Equation (1), *I* denotes the applied current, *M* refers to the total mass of the structural battery, and *V*(*t*) indicates the time‐dependent voltage profile during discharge.

To address the reporting protocols proposed by Greenhalgh et al.,^[^ ^18^ ^]^ we explicitly define our normalization basis and disclose a complete mass inventory for the cell. Table S2 (Supporting Information) has therefore been reorganized to map each constituent to the normalization categories. The LFP‐ and LTO‐based composite electrode layers (including PVDF and carbon black) contribute to both the active‐material mass and the active‐cell mass, and the CFRP laminate (serving as current collector and structural reinforcement), aluminum film, and glass‐fiber layers contribute only to the full‐cell mass (encapsulation/current‐collection and structural elements). The summed totals are as follows: active‐material mass = 2.271 g (35.7% of the full‐cell mass; from 78 wt.% LFP in the cathode and 82 wt.% LTO in the anode), active‐cell mass = 3.607 g (56.8%; comprising the composite positive electrode 1.476 g, composite negative electrode 1.365 g, PP/PE/PP separator 0.0668 g, and liquid electrolyte 0.6989 g), and full‐cell mass = 6.356 g (equal to the device mass reported in Table S2, Supporting Information).

**Figure 10:**
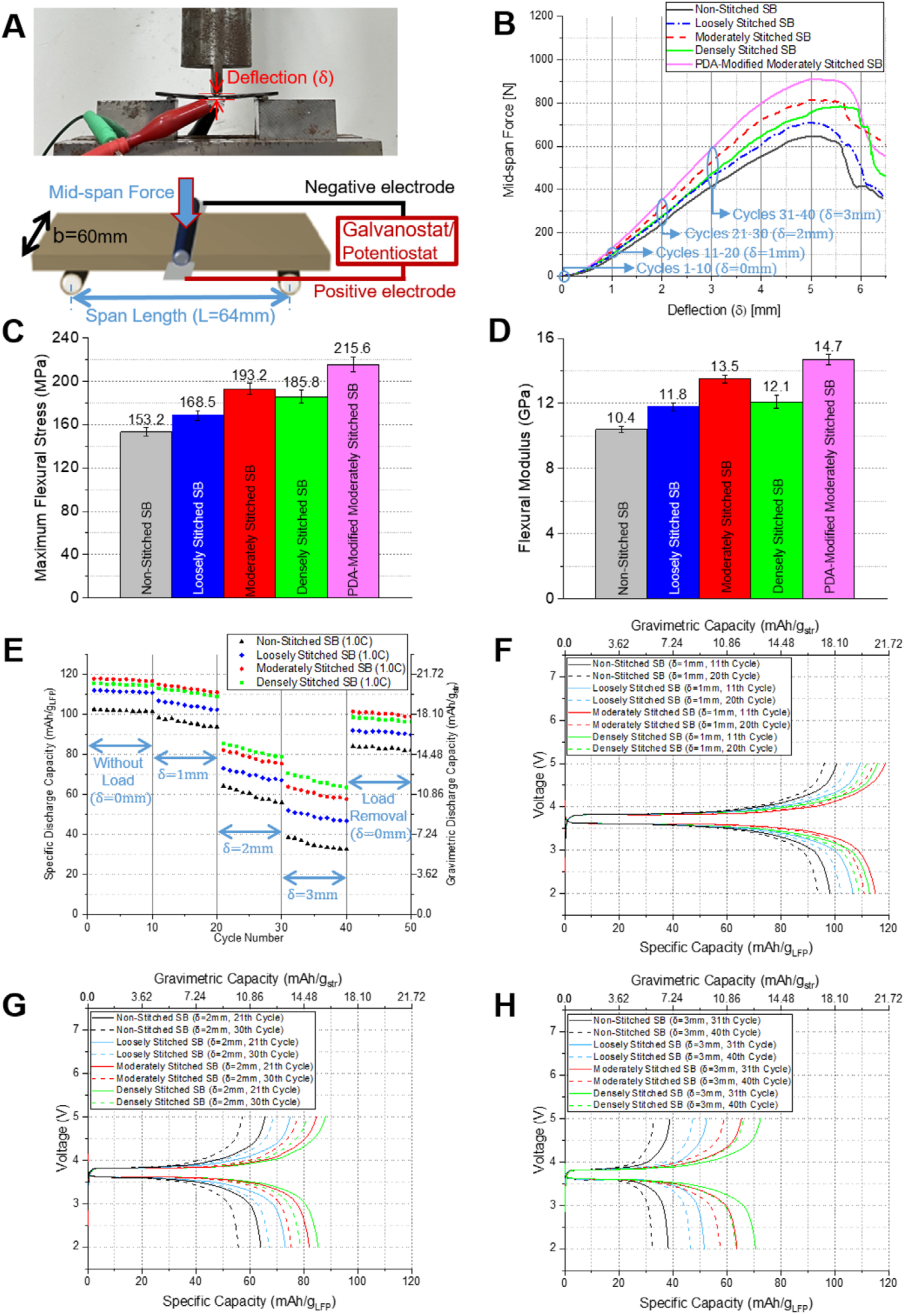
Mechanical and electrochemical response of structural batteries under bending. A) Schematic of the three‐point bending and in situ charge–discharge testing setup. B) Three‐point bending load–deflection curves. C) Maximum flexural stress. D) Flexural modulus. E) Evolution of discharge capacity under increasing deflection. Voltage profiles at deflection levels of F) 1 mm, G) 2 mm, and H) 3 mm, respectively.

Unless otherwise stated, all electrochemical data were acquired on vertically stacked, series‐connected structural‐battery laminates comprising two identical sub‐cells. All voltages reported in Figure 9 and **Figure** **10** are stack voltages measured across the laminate terminals. A single LFP∥LTO sub‐cell exhibits an open‐circuit voltage (OCV) of ≈1.7–1.9 V depending on state of charge;^[^ ^8^ ^]^ accordingly, the two‐cell laminate presents an OCV of ≈3.4–3.8 V (Figure 9B,D).

At a charge–discharge rate of 0.1C, the non‐stitched SB exhibited an initial capacity of 117.8 mAh g_LFP_^−1^, which gradually decreased to 111.8 mAh g_LFP_^−1^ by the 50th cycle, maintaining ≈95% of its original capacity (Figure 9A,B). Loosely Stitched SB showed improved initial and retained capacities of 127.4 and 121.7 mAh g_LFP_^−1^, respectively, maintaining a similar retention rate. The moderately stitched SB further enhanced initial and retained capacities to 134.1 and 128.3 mAh g_LFP_^−1^, respectively, while the densely Stitched SB demonstrated slightly lower capacities of 131.5 and 125.7 mAh g_LFP_^−1^ at initial and post‐50 cycles. Notably, capacity decay trends remained similar across all battery configurations, with ≈94–95% retention at higher C‐rates (1C), demonstrating consistency across cycling conditions (Figure 9C,D).

Gravimetric discharge capacities at 0.1C were determined to be 10.7 mAh g_str_^−1^ (non‐stitched SB), 11.5 mAh g_str_^−1^ (loosely stitched SB), 12.1 mAh g_str_^−1^ (moderately stitched SB), and 11.9 mAh g_str_^−1^ (densely stitched SB), translating to energy densities of 36.7, 39.7, 42.2, and 40.4 Wh kg^−1^, respectively (Figure 9A,B). Thus, stitching resulted in an approximate improvement range of 8.5–14.1% in discharge capacity and energy density relative to non‐stitched SB. Within the stitched series evaluated (stitch spacings 5, 3.33, and 2.5 mm; plus the unstitched control), the moderately stitched SB (3.33 mm spacing) exhibited the highest electrochemical performance within the studied range, attaining a gravimetric energy density of 42.2 Wh kg^−1^ at 0.1C (Figure 9E).

At a charge–discharge rate of 1C, the three stitched structural battery configurations exhibited enhanced performance compared to the non‐stitched SB, which delivered 9.3 mAh g_str_^−1^ and 32.6 Wh kg^−1^ (Figure 9C,D). The stitched variants achieved gravimetric discharge capacities ranging from 10.1 to 10.7 mAh g_str_^−1^ and corresponding energy densities between 35.4 and 37.2 Wh kg^−1^, representing an improvement of ≈9.2% to 15%. Among these, the moderately stitched SB yielded the highest performance, with 10.7 mAh g_str_^−1^ and 37.2 Wh kg^−1^, corresponding to a maximum capacity gain of 15% (Figure 9C,D). The capacity enhancement trend observed at 1C closely mirrored that at 0.1C, suggesting a consistent influence of stitching density across different rates. These results confirm that an optimal stitch spacing of ≈3.33 mm—between 2.5 and 5 mm—yields the highest energy density among the configurations evaluated.

Electrochemical impedance spectroscopy (EIS) was conducted to assess how variations in stitch density affect interfacial characteristics at the electrode–electrolyte boundary. The Nyquist plots shown in Figure 9F correspond to non‐stitched SB, loosely stitched SB, moderately stitched SB, and densely stitched SB configurations. An equivalent circuit model was utilized to extract quantitative resistance parameters. The high‐frequency intercept of the semicircle on the real axis represents the solution resistance (*R*_s_), which accounts for ion transport resistance through the electrolyte.^[^ ^19^ ^]^ The semicircle observed in the intermediate frequency region is indicative of charge‐transfer processes, represented by *R*_ct_, occurring across the electrode–electrolyte boundary.^[^ ^19^, ^20^ ^]^ At lower frequencies, the sloped region reflects lithium‐ion diffusion behavior, typically modeled as a Warburg impedance element.

Compared to the non‐stitched SB, all stitched variants demonstrated slightly reduced R_s_ values, suggesting that the stitching improves local contact between the electrode and electrolyte, thereby facilitating faster ion transport. Notably, all stitched structures exhibited significantly reduced *R*_ct_, with the order being moderately stitched SB < densely stitched SB < loosely stitched SB. This trend is consistent with the charge–discharge performance, indicating enhanced charge transfer kinetics due to reduced interfacial separation and improved electrode cohesion. The moderate stitch density likely balances reinforcement and damage, maintaining optimal contact without excessive disruption to the active surface. In contrast, densely stitched SB, despite showing better performance than loosely stitched SB, exhibited a slight increase in *R*_ct_ compared to the moderately stitched configuration, likely due to partial electrode damage or reduced active surface area induced by high stitch density.

Collectively, these observations support that through‐thickness stitching can concurrently reduce *R*_s_ and *R*_ct_, enhancing ion transport and interfacial kinetics. This multifunctional benefit of stitching contributes to simultaneous improvements in electrochemical and mechanical integrity of structural batteries.

Figure 9G presents the cycling behavior evaluated across 500 successive charge–discharge iterations conducted at a rate of 0.5C. The moderately stitched SB exhibited the highest initial discharge capacity at 127.3 mAh g_LFP_^−1^. After 300 cycles, the capacity decreased to 104.4 mAh g_LFP_^−1^, representing an 18% reduction. After 500 cycles, it further declined to 98 mAh g_LFP_^−1^, corresponding to a total reduction of 23% and a capacity retention of 77%. These results suggest robust structural stability and electrolyte containment, attributable to the composite encapsulation with polypropylene and aluminum films. All SB variants exhibited similar irreversible capacity loss trends; however, stitched samples consistently outperformed the non‐stitched SB across the entire cycling range.

The retention of long‐term electrochemical performance without significant electrolyte leakage implies that the sealing structure effectively prevents moisture and oxygen ingress. Given the sensitivity of lithium‐based electrolytes, such barrier integrity is critical for prolonged battery operation. Our findings indicate that these stitched structural batteries, especially with moderate stitch density, offer a promising route for realizing mechanically robust, high‐energy‐density systems suitable for extended service in practical applications.

Previous CFRP‐based structural battery designs often struggled to reduce electrode spacing without the use of external rigid casings, which added significant mass and consequently lowered overall energy density.^[^ ^1^, ^8^ ^]^ In contrast, our strategy employs a controlled stitch density in combination with a single‐layer CFRP prepreg shell, effectively maintaining minimal electrode separation while eliminating the need for heavy structural reinforcements. This design not only preserves mechanical integrity and electrochemical functionality, but also maximizes the proportion of active material per unit mass. As a result, our structural battery achieves markedly higher energy densities than those reported in most prior studies, highlighting the critical role of stitch optimization and shell minimization in multifunctional energy storage systems.

Although bipolar electrodes are theoretically advantageous for reducing overall mass in stacked‐series configurations, they were not adopted in this study due to practical limitations. Specifically, stitching through bipolar electrodes complicates the separation of electrochemical layers between the top and bottom surfaces, and maintaining effective moisture and oxygen barrier properties with liquid electrolytes becomes significantly more challenging. Therefore, CFRP‐based bipolar electrodes were excluded from this design.

Despite these constraints, the vertically stacked series‐connected architecture enabled the proposed structural battery to achieve a high output voltage of 5 V and superior energy density compared to most previously reported structural battery systems (Table S3,^[^ ^21^, ^22^, ^23^, ^24^, ^25^, ^26^, ^27^, ^28^, ^29^, ^30^, ^31^, ^32^, ^33^, ^34^, ^35^, ^36^, ^37^, ^38^, ^39^, ^40^, ^41^, ^42^, ^43^ ^]^ Supporting Information). This enhancement is primarily attributed to the vertical stacking configuration, which minimizes inter‐cell wiring length and associated electrical losses, while also reducing the dead space between cells. As a result, a greater proportion of the battery's total mass contributes directly to energy storage. Each cell is joined through interlaminar stitching and subsequently bonded, ensuring mechanical interfacial integrity across all electrode layers, regardless of their position within the laminate. Furthermore, the use of continuous fiber reinforcement simplifies the fabrication of large‐area structural batteries by providing both mechanical robustness and manufacturing scalability.

### 3.4 Effect of Stitch Architecture and Dopamine Functionalization on the Flexural Properties of Structural Batteries

To investigate the mechanical robustness and electrochemical reliability of structural batteries under bending deformation, a three‐point bending experiment was conducted in accordance with ASTM D790. All specimens were prepared with dimensions of 60 mm in width and 80 mm in length, and the span length was fixed at 64 mm (Figure 10A). A monotonic central load was applied at a rate of 1.3 mm min^−1^ to the mid‐span region while the corresponding center deflection (*δ*) was measured. The mechanical response, recorded as load–deflection curves, is presented in Figure 10B. Electrochemical cycling tests were concurrently performed during deformation stages: *δ* = 1 mm, *δ* = 2 mm, and *δ* = 3 mm, each followed by 10 consecutive galvanostatic charge–discharge cycles. Subsequently, after load removal (*δ *= 0 mm), an additional 10 cycles were performed to assess recovery characteristics.

The flexural stress (σ_*f*_) was computed using the following relation:

(2) $$\mathrm{Flexural}\;\mathrm{stress}\;\left(\sigma_{f}\right)=\frac{3\mathrm{PL}}{2bd^{2}}$$

where *P* is the applied mid‐span load (*N*), *L* is the support span (mm), *b* is the specimen width (mm), and *d* is the specimen thickness (mm). The maximum flexural stress derived using Equation (2) is summarized in Figure 10C. Likewise, the flexural modulus (*E_f_*) was obtained from the initial linear region of the load–deflection curve using:

(3) $$\mathrm{Flexural}\;\mathrm{modulus}\left(E_{f}\right)=\frac{L^{2}m}{4bd^{3}}$$

here, m represents the slope of the linear portion of the curve (N mm^−1^). The resulting flexural moduli are plotted in Figure 10D.

The non‐stitched SB exhibited σ_*f*_ = 153.2 MPa and *E_f_* = 10.4 GPa. With Loosely Stitched SB, values increased to σ_*f*_ = 168.5 MPa and *E_f_* = 11.8 GPa, corresponding to enhancements of ≈10% and 13.5%, respectively. Moderately stitched SB demonstrated a significant improvement, with σ_*f*_ = 193.2 MPa and *E_f_* = 13.5 GPa, representing increases of 26.1% and 29.8% over the unstitched case. Densely stitched SB reached σ_*f*_ = 185.8 MPa and *E_f_* = 12.1 GPa, yielding respective increases of 21.3% and 16.3% compared to non‐stitched SB (Figures 9, 10).

These results confirm that introducing a moderate stitch density can enhance the flexural performance of structural batteries. Improved interlaminar bonding due to stitching likely contributed to the increased stiffness and strength under bending loads. However, excessively dense stitching may lead to localized damage, as previously observed in SEM micrographs, where repeated needle penetrations compromise fiber continuity in the CFRP current collector. This degradation offsets the mechanical gains from enhanced bonding. Consequently, an overly high stitch density can ultimately reduce mechanical performance, a trend that aligns with prior findings in stitched composite laminates.^[^ ^44^, ^45^ ^]^ Taken together, the bending test results indicate that optimal stitch density, particularly the moderate configuration, provides a favorable balance between mechanical reinforcement and material integrity, thereby maximizing flexural strength without inducing excessive structural damage.

Building upon our previous work^[^ ^17^, ^46^ ^]^ on hybrid laminates and surface functionalization strategies, we applied a polydopamine (PDA) coating to enhance interfacial bonding between the aluminum film and the thin epoxy film in the structural battery system. The resulting structure, incorporating the PDA‐coated aluminum film, was integrated into the previously identified moderately stitched SB configuration, which exhibited the highest flexural performance. This improved variant is referred to as the PDA‐modified moderately stitched SB.

Carbon fibers typically possess high surface energy due to oxidative sizing treatments, enhancing epoxy adhesion. In contrast, untreated aluminum surfaces exhibit relatively low surface energy and chemically inert characteristics, resulting in inferior epoxy bonding without appropriate surface modifications. Thus, surface functionalization via PDA presents a viable strategy to improve the bonding performance of aluminum in hybrid composites.^[^ ^47^ ^]^

Mechanical evaluation using three‐point bending tests revealed that the PDA‐modified moderately stitched SB achieved a flexural strength (σ_*f*_) of 215.6 MPa and a flexural modulus (*E_f_*) of 14.6 GPa. Compared to its uncoated counterpart, which had σ_*f*_ = 193.2 MPa and *E_f_* = 13.5 GPa, this corresponds to respective increases of 11.6% and 8.9% (Figure 10C,D). This result affirms that PDA treatment significantly improves mechanical integrity by enhancing interfacial bonding between the epoxy and aluminum layers.

To interrogate the effect of aluminum functionalization on interfacial bonding, we initiated an interlaminar delamination at the laminate edge with a razor blade and peeled the aluminum barrier/current‐collector from the structural‐battery stack; the exposed aluminum surfaces were then examined by SEM (Figure S1, Supporting Information). The unfunctionalized aluminum exhibits a relatively smooth surface with negligible epoxy residue, indicating predominantly adhesive failure along the aluminum–epoxy film interface (Figure S1A, Supporting Information). In contrast, the polydopamine‐functionalized aluminum exhibits a uniform nodular coating with widespread adherent epoxy islands and torn polymer patches, which is characteristic of a shift toward cohesive failure within the PDA/adhesive interphase and thus a higher interfacial bond to aluminum (Figure S1B, Supporting Information).

By combining moderate stitching with PDA‐coated aluminum film, the resulting structural battery exhibited a 40.7% increase in flexural strength (215.6 MPa) and a 41.3% enhancement in stiffness (14.6 GPa) compared to the baseline non‐stitched SB. While these values are competitive with several previously reported structural batteries (Table S3, Supporting Information), such as those by Chen et al.^[^ ^5^ ^]^ (σ_*f*_ = 181 MPa, *E_f_* = 4.4 GPa), Han et al.^[^ ^48^ ^]^ (σ_*f*_ = 203 MPa, *E_f_* = 12.1 GPa), and Liu et al.^[^ ^49^ ^]^ (σ_*f*_ = 201 MPa, *E_f_* = 13.5 GPa), they are modest compared to high‐performance systems utilizing solid electrolytes. In studies on structural batteries employing solid electrolytes, Shi et al.^[^ ^15^ ^]^ reported σ_*f*_ and *E_f_* values of 234.1 MPa and 14.5 GPa, respectively, while Chen et al.^[^ ^50^ ^]^ observed significantly higher values of 584 MPa and 52.4 GPa.

Nevertheless, such high mechanical performances typically rely on solid‐state electrolyte systems, which impose design constraints and often face challenges in achieving effective electrochemical performance due to issues such as limited interfacial bonding between the electrode and solid electrolyte. In contrast, to the best of our knowledge, the structural battery proposed in this study exhibits one of the most competitive flexural properties among CFRP‐based structural batteries utilizing liquid electrolytes (Table S3, Supporting Information). The synergistic combination of moderate stitch density and PDA surface functionalization demonstrates a robust and multifunctional strategy for structural batteries, enabling simultaneous enhancement of mechanical integrity and electrochemical performance beyond conventional designs.

### 3.5 Electrochemical Performance of Structural Batteries under Bending Deflection

To assess electrochemical behavior under mechanical deformation, structural batteries were subjected to progressive flexural loading during charge–discharge cycling. As outlined in the experimental setup (Figure 10A), a central load was applied via an Instron testing system to induce controlled deflection levels of *δ* = 1, 2, and 3 mm. At each deflection stage, 10 consecutive galvanostatic cycles were conducted at a rate of 1C. Following the final deflection stage (*δ* = 3 mm), the mechanical load was fully removed, and a final set of 10 cycles was performed to evaluate the reversibility of electrochemical degradation.

Figure 10E presents the evolution of discharge capacity over 50 cycles, highlighting the impact of deflection amplitude on electrochemical performance for each stitching configuration. The non‐stitched SB exhibited an initial discharge capacity in the range of 101.3–102.4 mAh g_LFP_^−1^ at *δ* = 0 mm. However, with increasing deflection, capacity progressively declined: at *δ* = 1 mm (Figure 10F, black line), capacity dropped to 93.8–98.2 mAh g_LFP_^−1^ (4.2–7.5% reduction); at *δ* = 2 mm (Figure 10G, black line), capacity further decreased to 55.8–64 mAh g_LFP_^−1^ (37.5–44.9% reduction); and at *δ* = 3 mm (Figure 10H, black line), capacity declined sharply to 32.5–38.4 mAh g_LFP_^−1^, representing a 62.5–67.9% decrease relative to the initial state.

Despite the observed degradation, stitched batteries consistently outperformed the non‐stitched SB across all deformation levels. At *δ* = 0 mm, the moderately stitched SB achieved the highest capacity (116.7–117.8 mAh g_LFP_^−1^), which was ≈15–15.2% higher than the non‐stitched SB (Figure 10E). At *δ* = 1 mm, this trend persisted, with moderately stitched SB maintaining 111.9–115 mAh g_LFP_^−1^ (Figure 10F, red line), outperforming the non‐stitched SB by 17.1–19.3%. However, at larger deflections (*δ* = 2 mm), the densely stitched SB began to surpass the moderate configuration, achieving 78.8–85.3 mAh g_LFP_^−1^ (Figure 10G, green line), compared to 75.4–82.1 mAh g_LFP_^−1^ (Figure 10G, red line) for the moderately stitched counterpart. These values represented 28.3–34.9% and 33.3–41.2% improvements over the non‐stitched SB, respectively.

At the highest tested deflection (*δ* = 3 mm), the densely stitched SB retained the greatest capacity (63.4–70.7 mAh g_LFP_^−1^), outperforming both moderately stitched SB (57.6–63.8 mAh g_LFP_^−1^) and non‐stitched SB (32.5–38.4 mAh g_LFP_^−1^) by 10.1–10.8% and 84.1–95.1%, respectively (Figure 10H).

**Figure 11:**
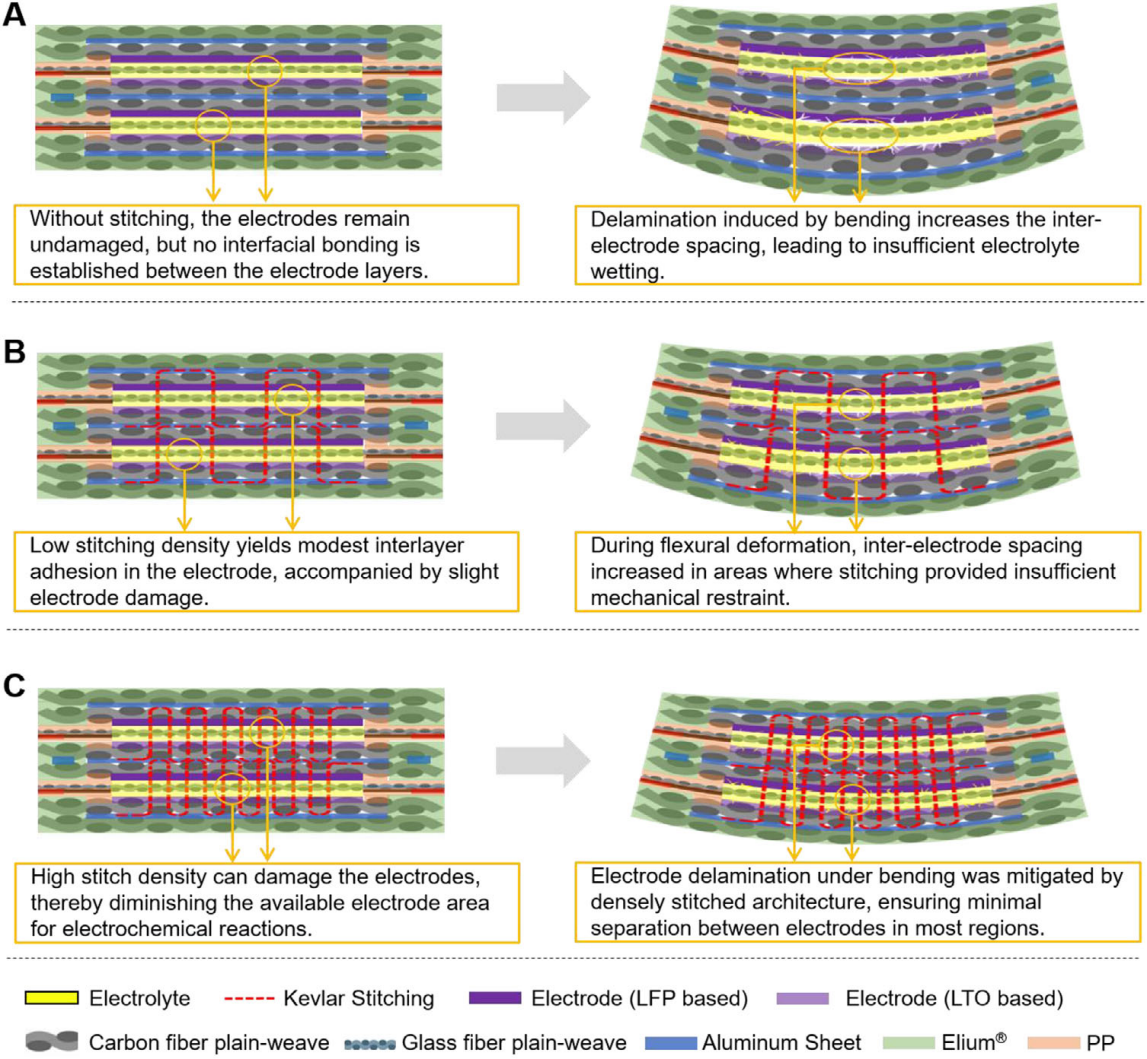
Comparison of inter‐electrode deformation behavior under flexural loading in structural batteries with varying stitching densities. A) Absence of stitching results in significant electrode separation. B) Loosely stitched structure provides moderate restraint. C) Dense stitching effectively limits electrode displacement.

To evaluate rate capability under mechanical load, we repeated the in situ three‐point bending experiments at two additional C‐rates (0.1C and 0.5C), keeping all other parameters identical to those used for the 1C tests in Figure 10 (deflection sequence *δ*: 0→1→2→3→0 mm). The resulting discharge‐capacity evolution is shown in Figure S2 (Supporting Information). As expected for LFP∥LTO cells, the absolute capacity increased at the lower rate (0.1C) and decreased at the higher rate (0.5C); however, its dependence on mechanical deflection closely mirrored the 1C results. In all cases, we observed i) a progressive capacity decrease with increasing *δ*, ii) partial recovery upon unloading to *δ* = 0 mm, and iii) improved retention with increasing stitch density. Stitched laminates consistently preserved higher capacity and exhibited smaller hysteresis, whereas the non‐stitched control showed the largest loss (Figure S2, Supporting Information vs Figure 10E). These findings underscore the increasing advantage of dense stitching in mitigating performance loss under severe deformation, likely due to its enhanced ability to preserve inter‐electrode proximity and alignment during bending (**Figure** **11**A–C).

Overall, the trend reveals that under mild deformation (*δ* ≤ 1 mm), moderately stitched SB performs best, likely due to minimal electrode damage and optimal bonding (Figure 11A,B). As deflection increases (*δ* ≥ 2 mm), densely stitched SB becomes more effective in maintaining interlaminar contact and preventing electrode delamination, thereby exhibiting superior capacity retention (Figure 11C). These results suggest that dense stitching plays a key role in mitigating interfacial degradation by restricting electrode separation during flexural stress.

Repeated cycling under flexural deformation also led to progressive capacity loss, with higher deflection inducing more severe degradation. This degradation is associated with stress‐driven microcrack formation in the electrode, separation between the electrode and current collector, as well as electrolyte intrusion into structurally compromised areas.^[^ ^15^, ^51^ ^]^ Such damage mechanisms have been reported in literature as principal failure modes in mechanically stressed structural batteries.^[^ ^1^, ^19^ ^]^

While stitching cannot fully prevent microcrack formation, it serves to suppress excessive delamination between electrode layers, improving charge transfer and aiding in capacity retention. This study demonstrates that a stitch architecture not only enhances mechanical resilience, but also enables structural batteries to retain electrochemical performance under cyclic deformation, highlighting the multifunctional advantages of through‐thickness stitching in energy‐storing composite systems.

To further investigate the electrochemical stability of the stitched architecture, an additional charge–discharge test was conducted following removal of the maximum deflection (*δ* = 3 mm). As shown in Figure 10E (Cycle 41–50), after the release of mechanical stress, the moderately stitched SB exhibited a recovered discharge capacity of 98.9–101.4 mAh g_LFP_^−1^, which exceeded that of the densely stitched SB (96.3–98.6 mAh g_LFP_^−1^), reinstating the original performance hierarchy observed in the undeformed state. Nevertheless, full recovery to the initial capacity (116.7–117.8 mAh g_LFP_^−1^) was not achieved, with the moderately stitched SB retaining ≈84.8–86% of its original capacity. In comparison, the non‐stitched SB retained only 81–82.1% (82.1–84 mAh g_LFP_^−1^), highlighting the enhanced resilience of the stitched configurations.

These results suggest that although stitching cannot entirely mitigate irreversible electrode damage caused by mechanical strain, it effectively limits structural delamination and interfacial separation, thus preserving a greater portion of the active electrode area for charge storage. This implies that through‐thickness stitching plays a critical role in suppressing mechanical degradation pathways (e.g., interlaminar separation, electrode dislocation), thereby improving energy retention following deformation.

Table S3 (Supporting Information) provides a systematic comparison of reported structural battery systems in terms of electrode chemistry, electrolyte type, specific capacity, energy density, and flexural performance. The energy density of 42.2 Wh kg^−1^ achieved by the moderately stitched SB in its pristine configuration is slightly below the highest values documented in literature, such as those reported by Huang et al.^[^ ^21^ ^]^ (≈43 Wh kg^−1^) and Singer et al.^[^ ^22^ ^]^ (exceeding 120 Wh kg^−1^), often realized through the use of solid electrolytes or novel electrode systems. Notably, this performance is attained through the use of standard carbon fiber electrodes coated with LiFePO_4_ and incorporating a liquid‐phase electrolyte, positioning our system competitively among carbon fiber‐reinforced polymer structural battery technologies.

For reference, Moyer et al.^[^ ^4^ ^]^ reported 35 Wh kg^−1^, Juntao et al.^[^ ^23^ ^]^ 26 Wh kg^−1^, and Jiang et al.^[^ ^24^ ^]^ 5.6 Wh kg^−1^ for systems that likewise employ carbon fiber current collectors with liquid or gel‐based electrolytes. These comparisons emphasize that the stitched structural battery presented in this work achieves superior electrochemical performance despite utilizing conventional materials, owing to the effectiveness of the stitching architecture in maintaining electrode alignment and structural integrity under load.

The structural battery developed in this research exhibits multifunctionality characterized by high energy density, superior bending strength, stable electrochemical capacity under mechanical deformation, and the capability for high‐voltage operation. The enhancement methods proposed, including stitching architectures, stacked cell configurations for high‐voltage outputs, electrolyte shielding through thermoplastic polymer encapsulation, and polydopamine surface treatments, are complementary rather than mutually exclusive. Thus, combining these approaches may provide viable solutions to certain shortcomings encountered in earlier structural battery studies. Especially, the stitching methodology can be synergistically combined with future innovations, such as damage‐tolerant electrodes, stitch‐compatible gel or soft polymer electrolytes, and other compliant interface materials.

### 3.6 Tensile Properties and Multifunctional Efficiency

**Figure 12:**
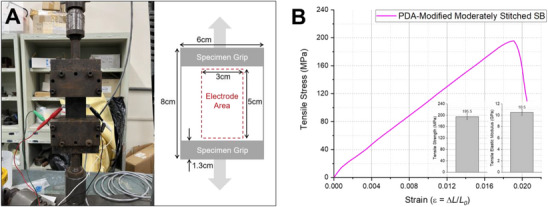
Tensile testing of the structural‐battery laminate. A) Test setup. B) Representative stress–strain curve of the PDA‐Modified moderately stitched SB; insets report mean tensile strength and modulus.

Tensile tests were conducted using bolted upper and lower grips (**Figure** **12**A) at a cross‐head rate of 2 mm min^−1^. The resulting stress–strain response of the PDA‐modified moderately stitched SB is shown in Figure 12B; the specimen delivered an ultimate tensile strength of 195.5 MPa and a tensile modulus of 10.5 GPa.

Our strength–modulus pair is comparable to values from carbon‐fiber‐based SBs using liquid or gel electrolytes, for example, Pint et al.^[^ ^4^ ^]^ (213 MPa, 1.8 GPa), Xu et al.^[^ ^30^ ^]^ (166 MPa, 5.6 GPa), and Ma et al.^[^ ^36^ ^]^ (155 MPa, 5.5 GPa), but lower than systems that demonstrated outstanding mechanical performance such as Sutrisnoh et al.^[^ ^40^ ^]^ (469 MPa, 71.6 GPa), Tian et al.^[^ ^41^ ^]^ (222 MPa, 22.4 GPa), and Wang et al.^[^ ^42^ ^]^ (299 MPa, 22.1 GPa).

Several design choices explain why our tensile properties are modest relative to the very best reports. First, the tensile coupon was intentionally configured to prioritize electrochemical area: the overall coupon was 6 cm × 8 cm with a 3 cm × 5 cm electrode window, so ≈31% of the surface area was devoted to electrodes, which necessarily reduced the Elium bonding area along the edges; a smaller electrode footprint is expected to raise the measured stiffness and strength. Second, to ensure long‐term electrochemical durability we introduced a PP perimeter barrier around the electrode zone. PP is an excellent moisture/oxygen barrier but adheres less strongly to carbon fibers than the Elium matrix, which can reduce interfacial strength in tensile loading.

In laminated structural batteries, the flexural modulus (*E*_f_) is lower than the tensile modulus (*E*_t_) because three‐point bending probes both axial bending stiffness and interlaminar‐shear compliance arising from the electrolyte/separator interleaves, whereas uniaxial tension is dominated by the load‐bearing carbon‐fiber tows. In liquid‐electrolyte stacks, the interleaf is not load‐bearing in shear, bending, or compression, so most bending resistance is carried by the CFRP facesheets while interfacial slip in the stack further depresses the measured flexural stiffness. By contrast, the tensile modulus reflects the axial stiffness of the fiber reinforcement and is comparatively insensitive to interleaf shear compliance, hence *E*_t_ > *E*_t_.

To benchmark multifunctional performance, we adopted the multifunctional efficiency (MFE) metric, widely used to co‐evaluate electrochemical and structural functions in structural energy‐storage devices. MFE is defined as the sum of an energy efficiency term (η_*e*_) and a structural efficiency term (η_*s*_): η_*mf*_ = η_*e*_ + η_*s*_, where η_*e*_ = Γ_*mf*_/Γ_*std*_ and η_*s*_ = *E_mf_*/*E_std_*. Following prior work, the baselines were set to a standard LFP Li‐ion battery Γ_*std*_ = 90 Wh kg^−1^ and an automotive‐grade unidirectional CFRP modulus *E_std_* = 128 GPa; within this framework η_*mf*_ > 1 indicates system‐level mass savings over a separated structure + battery solution.

Applying this to our representative specimen (PDA‐Modified moderately stitched SB: Γ_*mf*_ = 42.2 Wh kg^−1^; *E_mf_* = 10.5 GPa), we obtain η_*e*_ = 0.469, η_*s*_ = 0.082, and thus η_*mf*_ = 0.551. This value lies within the band reported for contemporary structural batteries: system‐level “multifunctional batteries” typically exhibit η_*mf*_ ≈ 0.1–0.9 with material‐level structural batteries often remain below unity due to the well‐known trade‐off between stiffness and energy density.

Reports of high multifunctional efficiency in the literature have often been realized by maximizing a single attribute rather than balancing electrochemical and structural functions (Table S3, Supporting Information). For instance, MnOx/Zn systems attain high specific energy but exhibit limited long‐term cycling stability.^[^ ^48^ ^]^ NMC/carbon‐based structural electrodes demonstrate exceptional stiffness at the laminate level, yet their electrochemical performance has not been rigorously validated, particularly under extended cycling.^[^ ^33^ ^]^ By contrast, an LCO‖NMC/graphite configuration is one of the few cases reported to combine elevated energy density with long‐term durability.^[^ ^36^ ^]^ Collectively, these studies tend to privilege one function at the expense of the other, underscoring the rarity of robust, sustained multifunctional performance.

In our study, MFE is used not as an absolute claim of system‐level mass savings but as a comparative figure of merit to quantify how effectively our interlaminar reinforcement strategy—dopamine‐functionalized aluminum interfaces combined with moderated through‐thickness stitching—balances energy density and stiffness. Although our η_*mf*_ = 0.551 is not the highest reported, it lies in the upper range of material‐level structural batteries to date and exceeds that of most laminate implementations using structural battery electrolytes. Many carbon‐fiber‐based multifunctional materials report η_*mf*_ ≈ 0.16–0.58, with only a few outliers approaching ≈0.88. These literature trends, synthesized in recent reviews and representative device reports, support our interpretation of η_*mf*_ = 0.551 as a comparatively strong, well‐balanced outcome rather than a single‐attribute extreme (see Table S3, Supporting Information for calculated comparisons).

## 4 Conclusion

This study presents a high‐voltage laminated structural battery that strategically integrates through‐thickness aramid stitching, selective thermoplastic polymer encapsulation, and vertical cell stacking to simultaneously optimize mechanical robustness and electrochemical performance. The tailored stitching architecture effectively reduced interlayer gaps and maintained uniform electrode spacing, enhancing ionic conductivity and capacity retention under both static and dynamic mechanical loads. Compared to the unstitched structural battery, the optimally stitched configuration achieved a 15% enhancement in energy density, reaching 42.2 Wh kg^−1^, and sustained 77% capacity retention over 500 cycles. Under bending deformation, stitched configurations exhibited up to 95% higher capacity retention than unstitched counterparts, validating their superior mechanical–electrochemical coupling. In addition, the incorporation of polypropylene barrier films and Elium thermoplastic matrix ensured long‐term electrolyte stability without compromising structural integrity. The use of a post‐injection electrolyte filling process enabled high‐temperature consolidation while preserving liquid electrolyte functionality. By vertically laminating two cells and connecting them in series, a high‐voltage (5 V) structural battery was fabricated, significantly reducing interconnect length and dead space compared to conventional planar designs. The combination of optimal stitch density and dopamine‐functionalized aluminum barriers enabled the configuration to maintain high flexural strength (215.6 MPa) and stiffness (14.7 GPa). This work offers a versatile and scalable design strategy for multifunctional energy storage, with broad applicability across next‐generation electric mobility platforms and high‐integrity pouch cell technologies that demand precise electrode alignment and long‐term electrolyte stability.

## Conflict of Interest

The authors declare no conflict of interest.

## Supporting information



Supporting Information

Supplemental Video 1

## Data Availability

Research data are not shared.
