# Supplementary material for: Tailored Stitching and Vertical Stacking for High‐Voltage Multifunctional Structural Batteries with Enhanced Electrochemical–Mechanical Coupling
Source: Adv Sci (Weinh). 2025 Oct 21;13(2):e14967. doi: 10.1002/advs.202514967 (PMC12786340; doi:10.1002/advs.202514967)
Supplement: Supplementary file 1 — Supporting Information [file ADVS-13-e14967-s002.docx]

*Supporting Information for:*

**Tailored Stitching and Vertical Stacking for High-Voltage Multifunctional Structural Batteries with Enhanced Electrochemical–Mechanical Coupling**

*Gilsu Park ^a^, Chun-Gon Kim ^b^, Heechul Kwon ^c^, Jayden Dongwoo Lee ^b^, Jinwoo Park ^d^, Yoonkook Son ^d^, Jong Guk Kim ^e^, Ji-Hun Cha ^c^**

^a^ Department of Aerospace Engineering, Chosun University, 309, Pilmun-daero, Dong-gu, Gwangju, Republic of Korea; 61452

^b^ Department of Aerospace Engineering, KAIST, 291, Daehak-ro, Yuseong-gu, Daejeon, Republic of Korea; 34141

^c^ School of Space Engineering Sciences, Kyungpook National University, 80, Daehak-ro, Buk-gu, Daegu, Republic of Korea; 41566

^d^ Department of Electrical Engineering, Chosun University, 309, Pilmun-daero, Dong-gu, Gwangju, Republic of Korea; 61452

^e^ Department of Advanced Energy Engineering, Chosun University, 309, Pilmun-daero, Dong-gu, Gwangju, Republic of Korea; 61452

*Corresponding author. E-mail: space_composites@knu.ac.kr

**Materials**

Electrochemically active cathode and anode powders, including lithium iron phosphate (LFP, EQ-Lib-LFPO-21), lithium titanate (LTO, EQ-Lib-LTO), and conductive carbon black (EQ-Lib-superP), were sourced from MTI Korea. The binder polymer, polyvinylidene fluoride (PVDF, molecular weight approximately 534,000), along with the solvent N-methyl-2-pyrrolidone (NMP, purity ≥ 99%, HPLC grade), were supplied by Sigma-Aldrich.

For electrolyte preparation, a commercially available 1 M solution of LiPF₆ dissolved in a 1:1 volumetric mixture of ethylene carbonate and diethyl carbonate (EC:DEC) was used, obtained from Welcos Korea. Dopamine hydrochloride (H8502) and tris(hydroxymethyl)aminomethane base (T1503), both analytical grade, were obtained from Sigma-Aldrich and used without further purification.

**Table S1**. **Configurations, features, and limitations in reported laminated structural battery composites.**

| References | Features and Components | Disadvantages |
| --- | --- | --- |
| Ladpli et al. [3] | Uses polymer resin to reinforce bonding between the CFRP sheet and the electrode stack, improving mechanical integrity.  The cathode and anode current collectors are metallic and are simply embedded within the CFRP, rather than forming an integrated structure. | A commercial metal current collector is used instead of a CFRP current collector.  Creating through-holes for rivet bonding and electrolyte injection reduces mechanical properties.  Polymer rivet bonding is used for interlayer adhesion, but the electrodes and outer composite skin panel are not structurally integrated.  The design does not specifically optimize CFRP current collectors, leading to reduced lightweight efficiency and mechanical performance. |
| Moyer et al. [4] | Carbon fiber plain-weave is used as the current collector, with LFP and graphite as active materials.  The ionic liquid electrolyte is pre-infused into the separator, followed by hand lay-up epoxy application and room-temperature curing.  Tensile strength: 213 MPa, tensile modulus: 1.8 GPa. | The ionic liquid electrolyte exhibits slightly lower electrochemical performance compared to conventional liquid electrolytes.  During vacuum curing, epoxy may mix with the electrolyte, infiltrate the electrode area, or evaporate, making fabrication highly challenging.  To preserve electrolyte performance, the curing temperature cannot be increased.  The lack of interlayer bonding to maintain narrow electrode spacing may lead to a reduction in energy storage capacity. |
| Chen et al. [5] | Carbon fiber plain-weave is used as the current collector, with MnO₂ and Zn as active materials.  The gel electrolyte is pre-infused into the separator, followed by hand lay-up epoxy application and room-temperature curing.  Tensile strength: 293 MPa, tensile modulus: 12.8 GPa. | The gel electrolyte exhibits slightly lower electrochemical performance than conventional liquid electrolytes.  During vacuum curing, epoxy may mix with the electrolyte, infiltrate the electrode area, or evaporate, making fabrication highly challenging.  To preserve electrolyte performance, the curing temperature cannot be increased.  The lack of interlayer bonding to maintain narrow electrode spacing may lead to a reduction in energy storage capacity. |
| Liu et al. [6] | Carbon fiber plain-weave is used as the current collector, with an MnO₂-coated cathode and Zn metal anode.  A structural battery utilizing a less oxygen/moisture-sensitive electrolyte (2M ZnSO₄ + 0.2M MnSO₄) is proposed.  The electrolyte is pre-infused into the separator, followed by hand lay-up epoxy application and room-temperature curing.  Tensile strength: 179 MPa. | An electrolyte less sensitive to oxygen and moisture exhibits slightly lower electrochemical performance than conventional liquid electrolytes.  The use of metallic materials as current collectors results in reduced mechanical properties and decreased weight reduction efficiency.  The lack of interlayer bonding to maintain narrow electrode spacing may lead to a reduction in energy storage capacity. |
| Dong et al. [7] | Carbon fiber plain-weave is used as the current collector, with LFP and graphite as active materials.  The conventional liquid electrolyte used in lithium-ion batteries is employed.  Structural reinforcement is achieved through vacuum-assisted high-temperature curing of epoxy resin, followed by the introduction of liquid electrolyte after solidification.  Tensile strength: 185 MPa, tensile modulus: 11.8 GPa. | The electrolyte shielding relies solely on epoxy polymer, but its moisture and oxygen permeability makes it challenging to preserve electrolyte performance in long-term environments.  The lack of interlayer bonding to maintain narrow electrode spacing may lead to a reduction in energy storage capacity. |
| Choi et al. [8] | Carbon fiber plain-weave is used as the current collector, with LFP and LTO as active materials.  The conventional liquid electrolyte used in lithium-ion batteries is employed.  Structural reinforcement is achieved through vacuum-assisted high-temperature curing of epoxy resin, followed by the introduction of liquid electrolyte after solidification.  To enhance the moisture and oxygen barrier, polypropylene (PP) is applied to the sides, while metal sheets are used on the top and bottom surfaces. | The design incorporates PP and metal sheets to enhance the moisture and oxygen barrier of the electrolyte, which slightly reduces mechanical properties and increases structural complexity.  Higher stiffness in the outer layer is required to prevent electrode delamination, leading to increased weight. |
| Fu et al. [9] | To achieve high-voltage output within a planar configuration, eight cells were connected in series.  The solid polymer electrolyte was in situ polymerized, directly incorporated into the prepreg composite without a pouch, and co-cured.  Cu/Al metal sheets serve as both current collectors and impact-resisting structural layers within the battery.  Elastic modulus: 25.3 GPa, impact resistance: 30 J | Since the current collector is metallic rather than CFRP, the mechanical performance is somewhat reduced.  Dead space between adjacent cells leads to a decrease in energy density.  The use of conductive metal interconnects for internal series connections further compromises the mechanical properties of the structure. |
| Xu et al. [10] | A high-voltage structural battery was developed through in-plane series integration of unit cells.  The structural battery composite cell with a PEO-based SBE matrix demonstrated stable capacity during repeated charge–discharge cycling under tensile loading up to 0.36% strain.  In-plane longitudinal modulus: 26.9 GPa, transverse modulus: 21.1 GPa, shear modulus of the laminate: 2.91GPa. | In-plane series configurations in structural batteries often suffer from increased ohmic losses due to long interconnects, inefficient volumetric utilization from inter-cell gaps, and compromised structural integrity arising from physical discontinuities.  They introduce fabrication complexity and uneven current distribution, limiting scalability and long-term reliability. |

$*$References are identical to those cited in the main text.

**Table S2**. **Mass inventory and structural power composites-normalization mapping.**

| Constituent | Weight fraction [%] | SPC* category | Material / composition |
| --- | --- | --- | --- |
| Positive electrode layers (composite) | 23.2 | AC* | LFP:PVDF:CB = 78:12:10 wt% |
| Negative electrode layers (composite) | 21.5 | AC* | LTO:PVDF:CB = 82:10:8 wt% |
| PP/PE/PP separator (trilayer) | 1.1 | AC* | PP/PE/PP film |
| Liquid electrolyte | 11.0 | AC* | 1 M LiPF₆ in EC/DEC |
| CFRP laminate (current collector + structure, incl. Elium® and PP edge barrier) | 38.1 | FC* | Plain-weave CF/Elium® + PP barrier |
| Woven glass fiber layers | 3.3 | FC* | Plain-weave GF |
| Aluminum film layers (outer encapsulation & inter-cell barrier) | 1.8 | FC* | Al |
| Active-material subtotal (AM = LFP + LTO solids only) | 35.7 | AM* | - |
| Active-cell subtotal (AC = electrodes + separator + electrolyte) | 56.8 | AC* | - |
| Full-cell total (FC = device mass used for specific energy) | 100.0 | FC* | - |

Structural power composites (SPC*); Active-material (AM*); Active-cell (AC*); Full-cell (FC*)

**
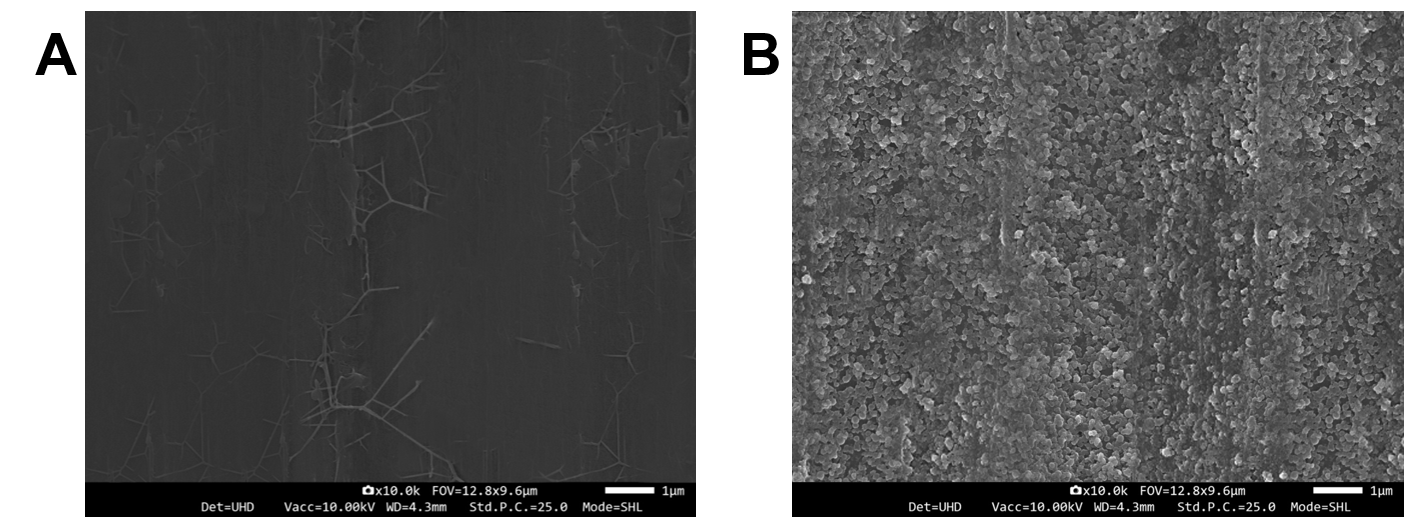
**

**Fig. S1 SEM fractography of aluminum film surfaces peeled from the structural-battery laminate. (A) Uncoated aluminum film surface. (B) Polydopamine-coated aluminum film surface.**


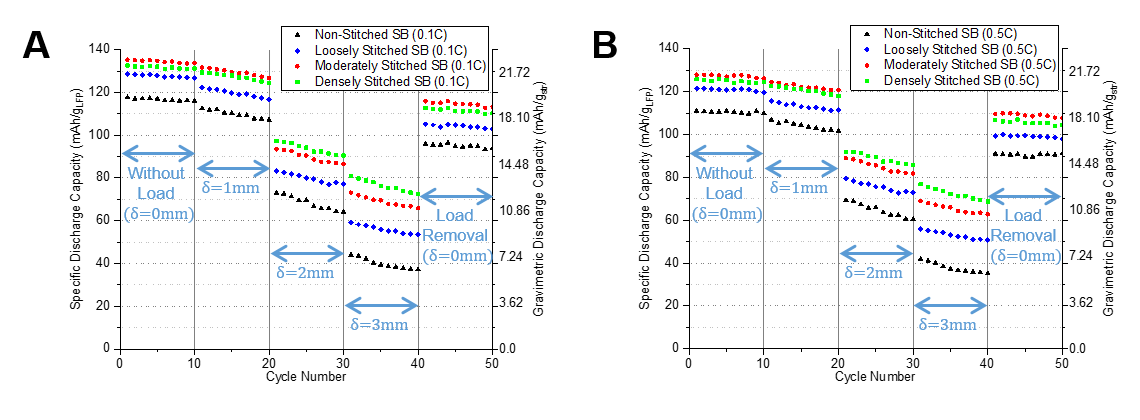


**Fig. S2 Mechanical and electrochemical response of structural batteries under bending. Evolution of discharge capacity in 0.1C (A) and 0.5 (B) under increasing deflection.**

**Table S3**. **Overview of multifunctional performance characteristics in carbon-fiber-integrated energy-storing composites.**

| Electrode configuration | Electrolyte system | Mechanical enhancement characteristics | Specific capacity  (mAh/g) | Energy density (Wh/kg) | Mechanical performance | Multifunctional efficiency | Cycle life retention | Cited work |
| --- | --- | --- | --- | --- | --- | --- | --- | --- |
| LFP ‖ Carbon  /  LTO ‖ Carbon | LiPF₆ dissolved in an EC/DEC | Stitched through-thickness reinforcement, dopamine coating,  CFRP used as current collector,  CFRP as outer reinforcement | 134 | 42.2 (based on full cell mass) | $\sigma_{f}$=215 MPa  $E_{f}$=14.7 GPa  $\sigma_{t}$=195 MPa  $E_{t}$=10.5 GPa | 0.551 | 77% retention after 500 cycles | This work |
| NMC ‖ Al /  Graphite ‖ Al | LiPF_6_ in EC/DMC/DEC | Interlocking rivet,  CFRP as outer reinforcement | - | - | - | - | 80% retention after 800 cycles | [3] |
| LFP ‖ Carbon  /  Graphite ‖ Carbon | 1M LiTFSI in EMIMBF_4_ | CFRP used as current collector,  CFRP as outer reinforcement | 30 | 35 (based on full cell mass) | $\sigma_{t}$=213 MPa  $E_{t}$=1.8 GPa | 0.403 | - | [4] |
| MnO_2_ ‖ Carbon  /  Zn ‖ Carbon | PVA/ZnCl_2_-MnSO_4_ | CFRP used as current collector,  CFRP as outer reinforcement,  Solid polymer-based electrolyte | 145 | - | $\sigma_{f}$=181 MPa  $E_{f}$=4.4 GPa  $\sigma_{t}$=293 MPa  $E_{t}$=181 GPa | - | 88% retention after 100 cycles | [5] |
| MnO_2_ ‖ Carbon  /  Zn sheet | 2M ZnSO_4_ + 0.2M MnSO_4_ | CFRP used as current collector,  CFRP as outer reinforcement | 155 | - | $\sigma_{f}$=230 MPa  $E_{f}$=12.9 GPa  $\sigma_{t}$=180 MPa  $E_{t}$=3.05 GPa | - | 85% retention after 300 cycles | [6] |
| LFP ‖ Carbon  /  Graphite ‖ Cu | 0.05 M LiPF_6_, | CFRP used as current collector,  CFRP as outer reinforcement | - | - | $\sigma_{f}$=197 MPa  $E_{f}$=5.1 GPa  $\sigma_{t}$=185 MPa  $E_{t}$=11.8 GPa | - | 99% retention after 30 cycles | [7] |
| LFP ‖ Carbon  /  LTO ‖ Carbon | LiPF_6_ in EC/DEC | CFRP used as current collector,  CFRP as outer reinforcement | 127 | 25 (based on full cell mass) | - | - | - | [8] |
| MgVO ‖ Carbon /  Zn ‖ Carbon | MAP/ZnSO_4_ (SPE) | CFRP used as current collector,  CFRP as outer reinforcement,  Solid polymer-based electrolyte | 344 | - | $\sigma_{f}$=234 MPa  $E_{f}$=14.5 GPa  $\sigma_{t}$=181 MPa  $E_{t}$=16.8 GPa | - | 82% retention after 1000 cycles | [15] |
| MnO_x_ ‖ Stainless /  Zn sheet | 2M ZnSO_4_ + 0.2M MnSO_4_ | CFRP used as current collector,  CFRP as outer reinforcement | 85 | 115 (based on full cell mass) | $\sigma_{f}$=203 MPa  $E_{f}$=12.1 GPa  $\sigma_{t}$=154 MPa  $E_{t}$=10.0 GPa | 1.356 | 51% retention after 500 cycles | [25] |
| PS ‖ Carbon  /  Li ‖ Carbon | BN/PVDF-LiTFSI | CFRP used as current collector,  Solid polymer-based electrolyte | - | 43 (based on full cell mass) | - | - | 81% retention after 200 cycles | [29] |
| NMC ‖ Al /  Graphite ‖ Cu | LiDFOB–LiBF₄ in DEC/FEC (SPE) | CFRP used as current collector,  CFRP as outer reinforcement,  Solid polymer-based electrolyte | 150 | 127 (based on full cell mass) | $\sigma_{f}$=184 MPa  $E_{f}$=21.7 GPa | - | 88% retention after 1000 cycles | [30] |
| LFP ‖ Carbon  /  Li foil | LiPF_6_ in EC/DMC/DEC | CFRP used as current collector,  CFRP as outer reinforcement | 112 | 26 (based on full cell mass) | - | - | - | [31] |
| NMC ‖ Carbon  /  LTO ‖ Carbon | PVDF/Li TFSI/Pyr13 TFSI (SPE) | CFRP used as current collector,  CFRP as outer reinforcement,  Solid polymer-based electrolyte | 35 | 5.6 (based on full cell mass) | $\sigma_{t}$=32 MPa  $E_{t}$=4.6 GPa | 0.098 | - | [32] |
| LFP ‖ Carbon  /  Graphite ‖ Carbon | Epoxy resin based polymer (LiTFSI: EMIM-TFSI) | CFRP used as current collector,  CFRP as outer reinforcement,  Solid polymer-based electrolyte | 83.6 | - | $\sigma_{f}$=205 MPa  $E_{f}$=19.9 GPa  $\sigma_{t}$=201 MPa | - | 99% retention after 100 cycles | [33] |
| CNT ‖ NiO_x_ ‖ Stainless  /  CNT ‖ FeO_x_ ‖ Stainless | PVA-based gel electrolyte containing 1 M KOH | CFRP used as current collector,  externally reinforced kevlar fiber | 130 | 1.4 (based on full cell mass) | - | - | - | [34] |
| LFP ‖ Carbon  /  Carbon | SBE/LiBoB-LiTf | CFRP used as current collector | - | 23.6 (based on full cell mass) | - | - | - | [35] |
| LFP ‖ Al  /  Carbon | Poly(ethoxylated dimethacrylate)-based electrolyte with LiBoB–LiTf complex in EC/PC mixture | CFRP used as current collector,  CFRP as outer reinforcement,  Solid polymer-based electrolyte | 14.7 | 41 (based on full cell mass) | $\sigma_{f}$=203 MPa  $E_{f}$=12.1 GPa  $E_{t}$=25.7 GPa | 0.656 | 40% retention after 500 cycles | [36] |
| LFP ‖ Carbon  /  Carbon | LiPF_6_ in EC/DEC | CFRP used as current collector | 131 | - | - | - | 88% retention after 300 cycles | [37] |
| NH_4_V_4_O_10_ ‖ Carbon  /  Zn sheet | PVHF/MXene-g-PMA (SPE) | CFRP used as current collector,  CFRP as outer reinforcement,  Solid polymer-based electrolyte | 110 | - | $\sigma_{f}$=584 MPa  $E_{f}$=52.4 GPa  $\sigma_{t}$=166 MPa  $E_{t}$=5.65 GPa | - | 95% retention after 500 cycles | [38] |
| LFP ‖ PAN ‖ Carbon  /  Graphite ‖ PAN ‖ Carbon | LiTFSI/EMIMBF₄ electrolyte | CFRP used as current collector,  CFRP as outer reinforcement,  PAN coating | - | - | - | - | 64% retention after 100 cycles | [39] |
| LFP ‖ Carbon  /  LTO ‖ Carbon | 1 M LiTFSI in EC : PC | CFRP used as current collector  CFRP as outer reinforcement | - | 33.4 (based on full cell mass) | $\sigma_{f}$=234 MPa  $\sigma_{t}$=261 MPa  $E_{t}$=34.2 GPa | 0.638 | 95% retention after 100 cycles | [40] |
| NMC ‖ Carbon  /  Carbon | 1 M LiPF₆ in EC:EMC | Ceramic based separator as inner reinforcement,  PEO binder | 56 | 30 (based on full cell mass) | - | - | 82% retention after 20 cycles | [41] |
| LFP ‖ AB ‖ Carbon  /  Graphite ‖ AB ‖ Carbon | LiPF₆ | CFRP used as current collector,  CFRP as outer reinforcement | 34 | 34.1 (based on full cell mass) | $\sigma_{t}$=119 MPa  $E_{t}$=13.1 GPa | 0.481 | 98.8% retention after 100 cycles | [42] |
| NMC ‖ Al  /  Graphite ‖ Cu | 3M LiFSI | SiO₂ filler,  Gel based electrolyte,  CFRP as outer reinforcement | - | 90 (based on full cell mass) | $\sigma_{t}$=36.5 MPa  $E_{t}$=11.7 GPa | 1.091 | - | [43] |
| LCO ‖ NMC  /  Graphite | 1 M LiPF6 in EC:DEC:EMC | Al film used as current collector,  CFRP as outer reinforcement,  PLA frame | - | 120 (based on full cell mass) | $\sigma_{f}$=123 MPa  $E_{f}$=24.5 GPa  $\sigma_{t}$=155 MPa  $E_{t}$=5.5 GPa | 1.376 | 92% retention after 500 cycles | [44] |
| LFP ‖ Carbon  /  Graphite | 1 M LiTFSI in DME:DOL | CFRP as outer reinforcement,  PEO binder | 76.5 | 0.632 (based on full cell mass) | $\sigma_{f}$=639 MPa  $E_{f}$=73.4 GPa  $\sigma_{t}$=488 MPa  $E_{t}$=12.6 GPa | 0.105 | 97.3% retention under 300 MPa load | [45] |
| LFP ‖ Carbon  /  Li | EP/GF-CPE (SN + LiTFSI + LiDFOB + FEC + PEGDA/E2BADMA) | EP interface toughening,  CFRP as outer reinforcement | 119 | 28 (based on full cell mass) | $\sigma_{f}$=349 MPa  $E_{f}$=29.7 GPa  $\sigma_{t}$=424 MPa  $E_{t}$=35.3 GPa | 0.587 | 83% retention after 100 cycles | [46] |
| LFP  /  Li | 1 M LiDFOB in EC:PC | Bis-A dimethacrylate (EBPADMA) incorporation,  LSPE film | 160 | - | $\sigma_{t}$=3.65 MPa | - | 61% retention after 500 cycles | [47] |
| LFP ‖ Carbon  /  Li | 1 M LiPF6 in EC:EMC | PEO binder | 155 | - | $\sigma_{t}$=469 MPa  $E_{t}$=71.6 GPa | - | 96.3% retention after 100 cycles | [48] |
| LFP ‖ Carbon  /  Carbon ‖ graphite | 1 M LiPF₆ in DEC:DMC:EC + VC | Porous resin electrolyte,  CFRP as outer reinforcement | 122 | 10 (based on full cell mass) | $\sigma_{f}$=120 MPa  $E_{f}$=15.3 GPa  $\sigma_{t}$=222 MPa  $E_{t}$=22.4 GPa | 0.286 | 91.9% retention after 100 cycles | [49] |
| MnO₂ ‖ Carbon  /  Zn-P ‖ Carbon | 1 M Zn(TFSI)₂ + 0.1 M Mn(TFSI)₂ in EC/PC | Epoxy based biphasic solid state electrolyte | 0.63 | 0.294 (based on full cell mass) | $\sigma_{f}$=194 MPa  $E_{f}$=11.1 GPa  $\sigma_{t}$=257 MPa  $E_{t}$=12.9 GPa | 0.104 | 82.5% retention after 1000 cycles | [50] |
| Few-layer graphene ‖ Carbon  /  Al mesh | EMImCl/AlCl₃ (1:1.3) | CFRP as outer reinforcement,  FLG coating | 124 | 8.3 (based on full cell mass) | $\sigma_{f}$=50.9 MPa  $\sigma_{t}$=299 MPa  $E_{t}$=22.1 GPa | 0.265 | 90% retention after 500 cycles of maximum | [51] |

$\sigma_{f}$: flexural strength; $E_{f}$: flexural modulus; $\sigma_{t}$: tensile strength; $E_{t}$: tensile modulus.

*Only the energy density normalized by the total structural mass is compared, not by the mass of the active materials.

$*$References are identical to those cited in the main text.
